# Three *foxg1* paralogues in lampreys and gnathostomes—brothers or cousins?

**DOI:** 10.3389/fcell.2023.1321317

**Published:** 2024-01-02

**Authors:** Galina V. Ermakova, Alexander V. Kucheryavyy, Nikolay S. Mugue, Aleksandr V. Mischenko, Andrey G. Zaraisky, Andrey V. Bayramov

**Affiliations:** ^1^ Shemyakin-Ovchinnikov Institute of Bioorganic Chemistry, Russian Academy of Sciences, Moscow, Russia; ^2^ Severtsov Institute of Ecology and Evolution, Russian Academy of Sciences, Moscow, Russia; ^3^ Russian Federal Research Institute of Fisheries and Oceanography (VNIRO), Moscow, Russia; ^4^ Koltzov Institute of Developmental Biology of Russian Academy of Sciences, Moscow, Russia; ^5^ Branch for the Freshwater Fisheries of the Russian Federal Research Institute of Fisheries and Oceanography, Moscow, Russia; ^6^ Pirogov Russian National Research Medical University, Moscow, Russia

**Keywords:** *foxg1*, lamprey, sterlet, sturgeon, forebrain development, telencephalon

## Abstract

*Foxg1* is a key regulator of the early development of the vertebrate forebrain and sensory organs. In this study, we describe for the first time three *foxg1* paralogues in lamprey, representative of one of two basally diverged lineages of vertebrates—the agnathans. We also first describe three *foxg1* genes in sterlet—representative of one of the evolutionarily ancient clades of gnathostomes. According to the analysis of local genomic synteny, three *foxg1* genes of agnathans and gnathostomes have a common origin as a result of two rounds of genomic duplications in the early evolution of vertebrates. At the same time, it is difficult to reliably establish pairwise orthology between *foxg1* genes of agnathans and gnathostomes based on the analysis of phylogeny and local genomic synteny, as well as our studies of the spatiotemporal expression of *foxg1* genes in the river lamprey *Lampetra fluviatilis* and the sterlet *Acipenser ruthenus*. Thus, the appearance of three *foxg1* paralogues in agnathans and gnathostomes could have occurred either as a result of two rounds of duplication of the vertebrate common ancestor genome (2R hypothesis) or as a result of the first common round followed by subsequent independent polyploidizations in two evolutionary lineages (1R hypothesis).

## Introduction

*Foxg1*, encoding a forkhead-binding domain (FBD)-containing transcription factor, plays a key role in the development of the telencephalon, a unique part of the forebrain of vertebrates. Disturbances in *foxg1* expression result in developmental abnormalities and a reduction in forebrain size (Xuan et al., 1995). In humans, intra- and intergenic mutations resulting in loss of function or altered expression of *FOXG1* are named FOXG1 syndrome (Wong et al., 2019; Wong et al., 2023; Craig et al., 2020). In addition to its function in the telencephalon, *foxg1* also plays a fundamental role in the development of the inner ear (Ding et al., 2020). A detailed overview of the history of research on *foxg1* and its functions and regulation in early vertebrate development was provided by Kumamoto and Hanashima (Kumamoto and Hanashima, 2017).

*Foxg1* gene(s) have been described in all groups of vertebrates, as well as invertebrates. The *foxg1* homologue in *Drosophila*, the *slp1/2* gene, is expressed in the head region of the embryo (Grossniklaus et al., 1994). In hemichordates, *foxg1* is expressed in a gradient manner in the proboscis with a maximum at its anterior end (Pani et al., 2012). In the ascidian embryo, *foxg* expression is described in the anterior neural plate boundary and specifies sensory neurons (Liu and Satou, 2019). In the lancelet, *foxg1* is first expressed in the anterior part of the first somite and later, on the third day of development, in individual cells of the cerebral vesicle (Toresson et al., 1998). In the adult lancelet, *foxg1* is expressed in a broad anterior domain that occupies a large part of the cerebral vesicle, which, according to the authors, is similar in pattern to the earliest expression of *foxg1* in E8.5 mouse embryos before the telencephalon subdivides into paired vesicles (Benito-Gutiérrez et al., 2021).

In lampreys, as representatives of jawless vertebrates, the expression of *foxg1* was described, and its heterochrony was shown in comparison with homologues in gnathostomes (Ermakova et al., 2019; Higuchi et al., 2019). The expression domain of *foxg1* in the forebrain was also described in hagfish, which, together with the expression of other marker genes, *pax6* and *emxB*, indicates the homology of this region with the telencephalon of gnathostomes (Sugahara et al., 2016).

In the evolutionary lineage of gnathostomes, *foxg1* genes have been described in representatives of all groups, and historically, by default, most gnathostomes (with the exception of teleosts) have been assumed to have a single *foxg1* gene. Only recently were three *foxg1* genes described in sharks (Hara et al., 2018).

In the present study, using the available version of the sea lamprey *Petromyzon marinus* genome as a basis, we revealed three *foxg1* genes in lampreys and described their expression. We also described the *foxg1* genes in sterlet, a representative of one of the early-diverging lineage of bony fish—Chondrostei (sturgeons). Since sturgeons are characterized by polyploidy (Redmond et al., 2023), five *foxg1* paralogues, which arose as a result of specific duplication in the ancestors of this group, were found in sterlet. Two pairs of these paralogues show very close homology (nucleotide sequence identity of more than 90%), which was confirmed by phylogenetic analysis. Because of such high similarity, these two additionally duplicated sister copies are not distinguishable by *in situ* hybridisation (ISH), which we used to study the dynamics of the spatial expression of *foxg1*. Thus, regarding *foxg1* expression in sturgeons, as in lampreys and sharks, there appear to be three *foxg1* genes, two of which are represented by almost identical duplicated copies.

Assuming the presence of three *foxg1* genes in cyclostomes (lampreys) and basal gnathostomes (cartilaginous fish, sturgeons, and bony fish), we attempted to identify the orthology of *foxg1* genes between these lineages that diverged early in vertebrate evolution. Such a search seems relevant in the context of studying the early evolutionary history of the vertebrate genome and could help establish the timing of whole-genome duplications (WGDs). Currently, it is generally accepted that the ancestral genome of vertebrates underwent at least two rounds of WGD, but the question of whether these duplications were common to cyclostomes and gnathostomes or occurred independently in them after their split is still debatable. The classic model proposed by S. Ohno (Ohno, 1970) includes the scenario of two rounds of WGD before the split of cyclostomes and gnathostomes. At the same time, in recent years, based on the analysis of high-throughput genomic sequencing data, a number of alternative hypotheses have been proposed, suggesting one common and one or more independent rounds of WGD for cyclostomes and gnathostomes (Smith and Keinath, 2015; Sacerdot et al., 2018; Smith et al., 2018; Simakov et al., 2020; Nakatani et al., 2021). In the context of this task, gene families containing three or more paralogues that arose as a result of two rounds of duplications and still preserved in cyclostomes and gnathostomes are of great interest since assessment of their orthology can provide additional arguments in favour of one of the scenarios.

Evidence that genes originated as a result of duplications in vertebrates could include the presence of only one copy in their closest relatives—invertebrate chordates (lancelets and tunicates) and hemichordates. A classic example here is *Hox* genes, one cluster of which is described in the lancelet, six in lampreys and four in gnathostomes (Parker et al., 2019). A similar result was reported for the *Noggin* genes, represented by one copy in lancelets and tunicates, four copies in lampreys and three copies in some gnathostomes (Ermakova et al., 2020). After confirming the origin of the genes as a result of duplication in ancestral vertebrates, to establish the timing of duplication and their unity or independence, it is necessary to establish the paired orthology of the genes under study in the lineages of cyclostomes and gnathostomes. The presence of at least three significant orthologues in both lineages indicates that they were more likely to have arisen because of two common rounds of duplications than because of independent ones. The phylogenetic affinity of gene/protein sequences, local genomic synteny (common neighbouring genes), common features of expression patterns indicating homology of regulatory elements, and functional properties may serve as evidence of orthology. For the *Noggin* genes, a combination of these analyses showed that before the split of the cyclostomes and gnathostomes, at least three different *Noggin* genes appeared in ancestral vertebrates, suggesting two rounds of common genome duplications (Ermakova et al., 2020). Individually, these tests may not give an unambiguous result, so it is important to carry them out together. Thus, in phylogenetic analysis, it has been repeatedly noted that proteins of lampreys, due to the acquired characteristics of their amino acid composition, are often more confidently grouped with each other than with orthologues of gnathostomes (the so-called “lamprey dialect”) (Onimaru and Kuraku, 2018).

In the present work, we analysed the phylogeny and synteny of the *foxg1* genes of cyclostomes and gnathostomes using available genomic databases, examined the expression patterns of three *foxg1* genes in lamprey and sterlet for the first time, and attempted to evaluate the orthology of the *foxg1* genes of agnathans and gnathostomes. As a result, we did not find strong evidence for pairwise orthology of the *foxg1* genes in these two evolutionary lineages. The analysis of the probable timing of *foxg1* duplications suggests that the second duplication, which led to the appearance of three copies of *foxg1*, occurred on similar time horizons in cyclostomes and gnathostomes. This suggests that the *foxg1* genes in both groups originated from ancestral rounds of WGD rather than being duplicated later. According to existing estimates, the second rounds of genomic polyploidization in the cyclostome and gnathostome lineages also occurred at similar time horizons, approximately 450–460 million years ago (Marlétaz et al., 2023). Because of this, it is not possible to reliably establish whether this round in the two lineages was common or independent. As a result, the observed phylogeny, synteny and expression patterns of *foxg1* genes can be explained by the 1R or 2R WGD hypothesis.

## Results

### Three *foxg1* genes are the basic set for vertebrates

Our analysis of the available current version of the genome of the sea lamprey *P*. *marinus* (https://www.ncbi.nlm.nih.gov/datasets/genome/GCF_010993605.1/) revealed the presence of three paralogues of the *foxg1* gene on chromosomes 17, 29 and 31. Homologous sequences were also found in the genome sequences of the arctic lamprey *Lethenteron camtschaticum* (GenBank: WFAB01000304.1, WFAB01000071.1, WFAB01000203.1; https://www.ncbi.nlm.nih.gov/datasets/genome/GCA_018977245.1/).

**FIGURE 1:**
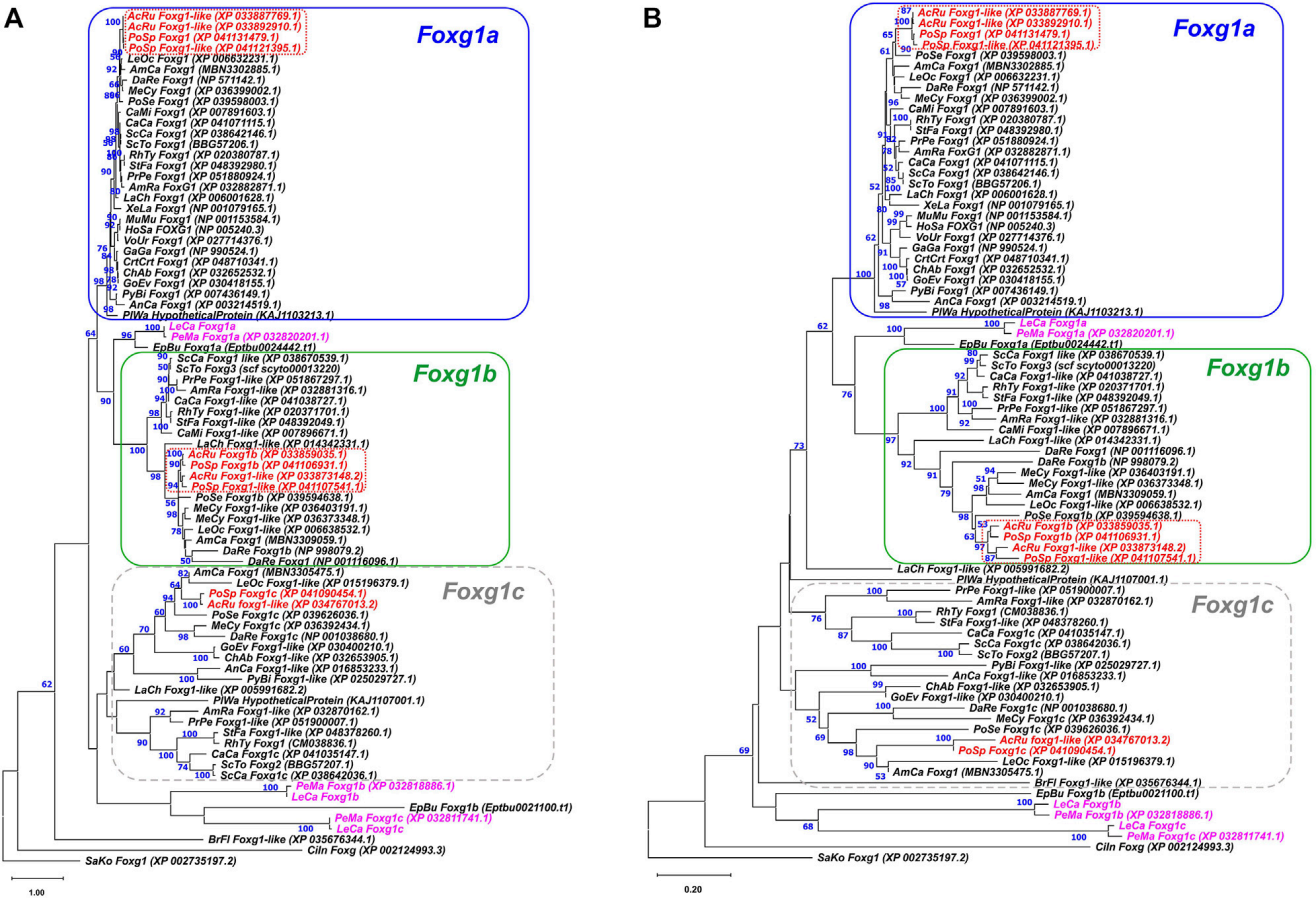
ML **(A)** and NJ **(B)** phylogenetic trees of Foxg1 proteins of vertebrates. Lamprey Foxg1 are violet, sturgeon Foxg1 are red. Bootstraps values > 50 are shown.

To assess the relationships of the detected sequences with the *foxg1* genes of gnathostomes, ML and NJ phylogenetic analyses of the amino acid sequences of proteins encoded by these genes were performed (Figure 1). Foxg1 sequences of gnathostomes were taken from available genome databases, and the sample included representatives of all described groups—cartilaginous fish (*Callorhinchus*, rays, skates and sharks), chondrosteans (sterlet, *Polyodon*), ray-finned and lobe-finned fish, amphibians, reptiles, birds and mammals. The analysis also included the *foxg1* genes of the closest relatives of vertebrates—lancelets, tunicates and hemichordates.

The search for sequences for analysis showed that the presence of one *Foxg1* gene observed in amphibians, birds, mammals and some reptiles is not a rule for vertebrates as a whole. In cartilaginous, sturgeon and bony fish, as a rule, three *foxg1* genes are present. Some representatives have two remaining genes (*Callorhinchus*), and teleosts, on the contrary, due to the additional round of teleostei-specific WGD (TS-WGD), have four *foxg1* paralogues. Since all the analysed closest relatives of vertebrates had one *foxg1* gene, it seems logical to assume that three vertebrate *foxg1* genes appeared as a result of two rounds of WGD. The names of proteins listed in the databases were saved during the analysis.

The phylogenetic trees of amino acid sequences of Foxg1 proteins constructed by the ML and NJ methods showed confident clustering of Foxg1a in gnathostomes, which includes the only Foxg1 proteins preserved in amphibians, birds and mammals. In databases, these proteins/genes are most often given the name Foxg1. Foxg1b proteins also cluster relatively confidently. In databases, these proteins/genes are often called Foxg1-like, and we assign the index “b” to them according to the genomes of sturgeon and bony fish. Foxg1c of gnathostomes represent a less monolithic group, in which subgroups of proteins of cartilaginous fish and sturgeons/bony fish are distinguished, and genes of coelacanths and reptiles are less confidently clustered with them. Separately, it should be noted that the “c” index for some genes/proteins of this group is present in the databases of cartilaginous, sturgeon and bony fish. To avoid further confusion, we tend to use these names instead of those given in the paper of Hara and colleagues (Hara et al., 2018), i.e., with the indexes FoxG1, FoxG2, and FoxG3 (where FoxG1 = Foxg1a, Foxg2 = Foxg1c, and FoxG3 = Foxg1b).

SaKo—*Saccoglossus kowalevskii*; CiIn—*Ciona intestinalis*; BrFl—*Branchiostoma floridae*; PeMa—*P. marinus*; LeCa—L. camtschaticum; EpBu—*Eptatretus burgeri*; CaMi—*Callorhinchus milii*; PrPe—*Pristis pectinata*; AmRa—Amblyraja radiata; ScCa—*Scyliorhinus canicula*; ScTo—Scyliorhinus torazame; RhTy—Rhincodon typus; CaCa—*Carcharodon carcharias*; AcRu—*Acipenser ruthenus*; PoSp—*Polyodon spathula*; PoSe—Polypterus senegalus; LeOc—*Lepisosteus oculatus*; AmCa—*Amia calva*; MeCy—Megalops cypronoides; DaRe—*Danio rerio*; LaCh—*Latimeria chalumnae*; AnCa—*Anolis carolinensis*; XeLa—*Xenopus laevis*; MuMu—*Mus musculus*; HoSa—*Homo sapiens*; StFa—Stegostoma fasciatum; GoEv—Gopherus evgoodei; ChAb—Chelonoidis abingdonii; PyBi—Python bivittatus; PlWa—*Pleurodeles waltl*; CrtCrt—*Caretta caretta*; GaGa—Gallus gallus; VoUr - *Vombatus ursinus*.

As seen from the structure of the phylogenetic trees (Figure 1; Supplementary Figure S7), three *foxg1* genes of lampreys and two genes of the hagfish *E*. *burgeri* cluster relatively reliably with each other but do not form confident pairs with genes of gnathostomes (which would be the first evidence of orthology). One of the lamprey and hagfish genes is closer to the *foxg1a* and *foxg1b* of gnathostomes, and the other two lamprey genes and the hagfish gene lie separately on the tree, closer to the *Branchiostoma*, *Ciona* and *Saccoglossus* genes. Due to the lack of pronounced pairwise phylogenetic clustering of the *foxg1* genes of cyclostomes and gnathostomes, the genes of cyclostomes were assigned the indices “α”,”β” and “γ”.

As an additional attempt to search for pairwise homology of Foxg1 proteins of agnathans and gnathostomes, an unrooted ML tree was constructed containing only proteins of the main groups of vertebrates in which several *foxg1* paralogues were found—lampreys, hagfish, sharks, spotted gar (representative of bony fish before the TS-WGD) and coelacanths (the evolutionary branch leading to terrestrial vertebrates) (Supplementary Figure S1). The results of this analysis confirmed the lack of reliable pairwise clustering of Foxg1 proteins in cyclostomes and gnathostomes.

To confirm the Foxg1 orthology in lampreys and hagfish, an unrooted ML tree with only agnathan proteins was constructed, according to which two Foxg1 proteins of hagfish confidently correspond to the Foxg1α and Foxg1γ proteins of lampreys.

The results of the phylogenetic analysis show that the Foxg1 proteins of agnathans and gnathostomes cluster quite confidently within each group, but no clear orthology of Foxg1 proteins can be traced between these evolutionary lineages.

In such a situation, it seems appropriate to analyse the local genomic synteny of *foxg1* and identify common genes in the vicinity of *foxg1* in representatives of different groups of vertebrates, which may provide evidence of their common origin.

**FIGURE 2:**
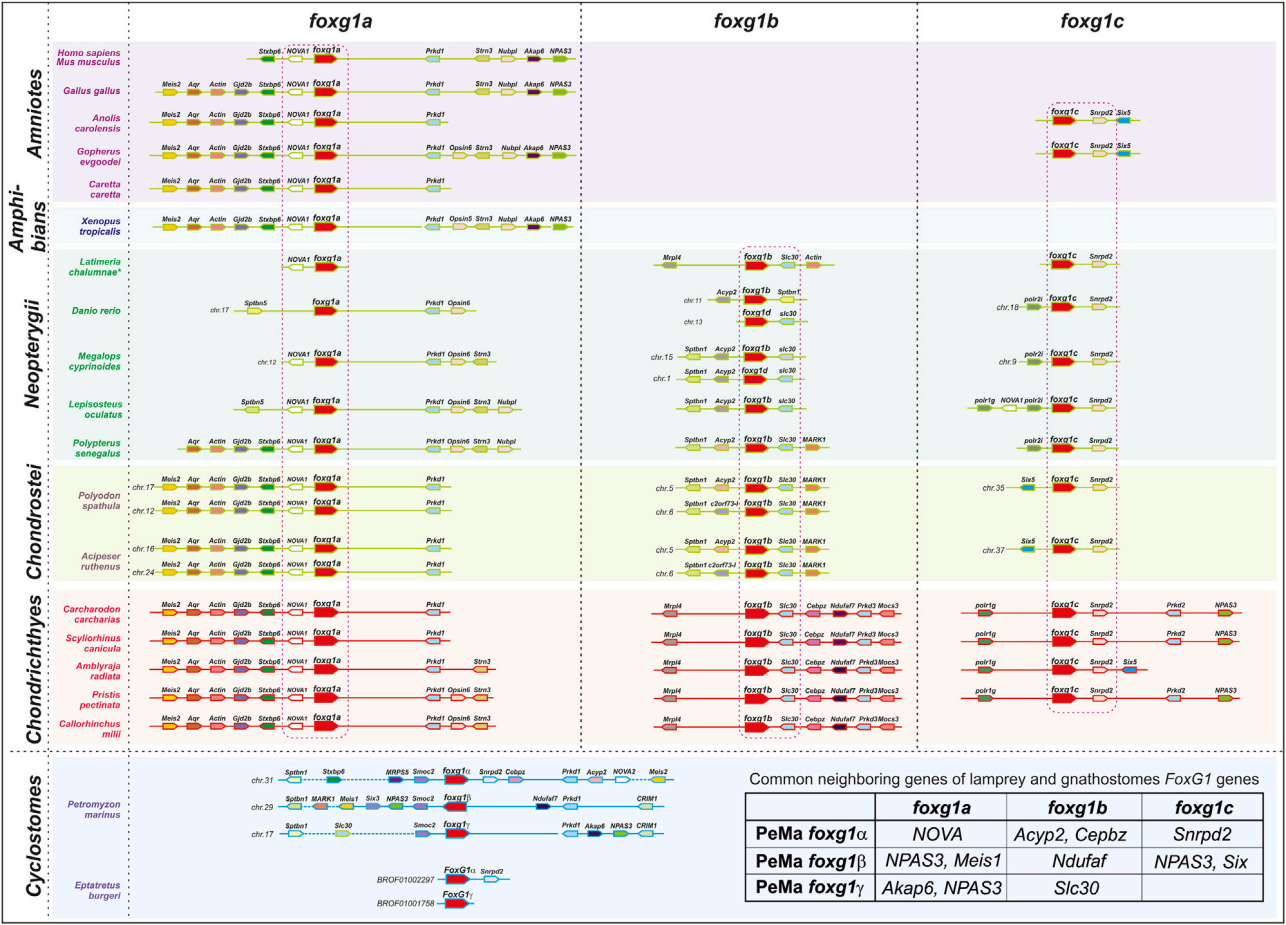
Analysis of local genomic synteny of vertebrate *foxg1* genes.

The results from the analysis of *foxg1* synteny in vertebrates are presented in Figure 2. It can be seen that each of the *foxg1* genes of gnathostomes has in its environment a number of unique genes, including one nearest neighbour—for *foxg1a*, these are homologues of the neuron-specific RNA-binding protein-coding gene *NOVA1*, for *foxg1b*—homologues of the zinc-transporter gene *slc30*, and for *foxg1c*—homologues of the small nuclear ribonucleoprotein D2 *snrpd2*. The presence of such reliable witness genes allows us to confidently classify *foxg1* genes in gnathostomes. In the vicinity of the *foxg1* genes of lampreys, many of the genes homologous to the *foxg1* neighbours of gnathostomes are found, but the lists of neighbours of the *foxg1* genes of lamprey overlap, preventing us from unambiguously determining the orthology between *foxg1* of lampreys and gnathostomes. The level of assembly of the current version of the hagfish genome [https://transcriptome.riken.jp/squalomix/blast/; *E*. *burgeri* genome assembly (Eburgeri_v1)] does not yet allow us to use it for confident analysis of genomic synteny; thus far, we can only confirm the orthology of the *foxg1α* genes of lampreys and hagfish, already observed in phylogenetic analysis.

Thus, in general, analysis of local genomic synteny of *foxg1* genes yields results similar to those of phylogenetic analysis. The *foxg1* genes of gnathostomes are confidently divided into three groups of paralogues: *foxg1a, foxg1b*, and *foxg1c*. At the same time, each of the *foxg1* genes in cyclostomes shares common neighbours with several *foxg1* genes in gnathostomes (common neighbour genes are shown in the table in Figure 2). This fact, indicating the common origin of the genes under consideration, makes it difficult to identify pairs of orthologues among them.

The next stage of the search for orthology may be a comparison of expression patterns of potential orthologues in different groups, which reflects the similarity or difference in regulatory elements, since in WGD, duplication occurs not only of open reading frame (ORF) genes but also of all regulatory elements. Although as a result of subsequent subfunctionalization, the spatial patterns of daughter genes may change, some of their individual features characteristic of the ancestral gene may be preserved and indicate the common origin of the genes in question (that is, their orthology).

### Dynamics and features of spatial expression of *foxg1* genes in river lamprey (*L. fluviatilis*)

**FIGURE 3:**
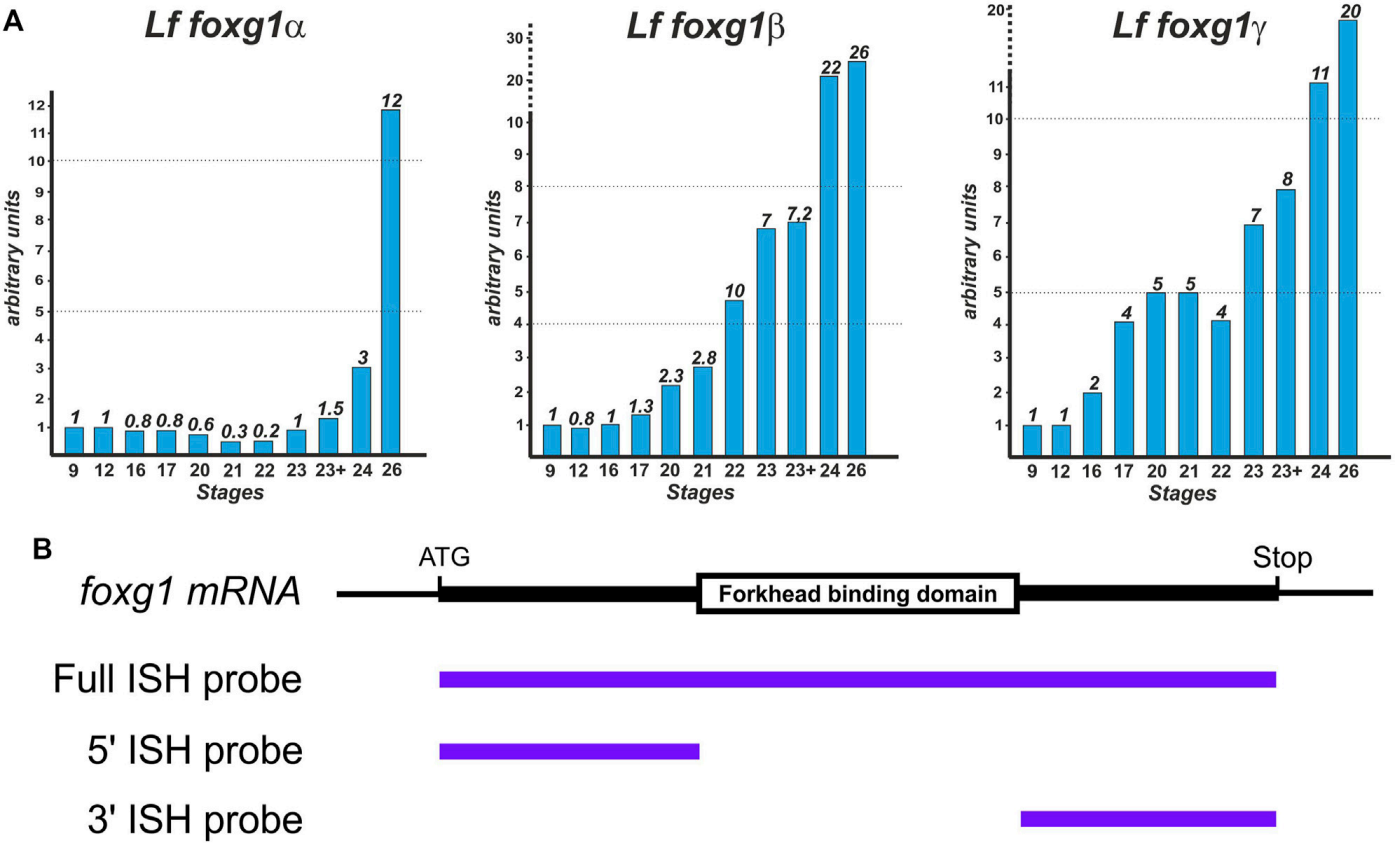
Dynamics of *foxg1* gene expression in *L. fluviatilis* (studied by RT‒PCR) **(A)** and scheme of probes for ISH **(B)**.

The temporal dynamics of *foxg1* gene expression in lampreys were examined by RT‒PCR (Figure 3A).

The expression profiles of all three *foxg1* genes in the river lamprey have common features: a low level at an early stage with a subsequent increase, which is observed later in the *foxg1β* and *foxg1γ* genes than in *foxg1α* (Figure 3A).

A study of the spatial expression of river lamprey *foxg1* genes was carried out using the whole-mount *in situ* hybridization (ISH) method at a series of early developmental stages—from the early neurula stage (stage 17 according to Tahara, 1988) to the ammocoete stage (stage 30). To synthesize ISH probes, cDNAs of river lamprey *foxg1* genes were obtained: a probe for the *foxg1α* gene with a size of 1470 bp and a probe for the *foxg1γ* gene with a size of 1500 bp, containing almost complete cDNAs of the genes under study. We previously obtained the full-length cDNA of the *foxg1β* gene, 1350 bp in size (Ermakova et al., 2019).

All three paralogues of *foxg1* contain a highly conserved FBD domain: the nucleotide identity of the FBD domains for *foxg1α* and *foxg1β* genes is 95%, and for *foxg1α* and *foxg1γ* genes, it is 93%. The presence of such conserved regions can lead to cross-hybridization of antisense ISH probes with mRNA of different *foxg1* genes. To confirm the specificity of the observed expression patterns, two additional probes complementary to the 5′ and 3′ regions of cDNA were also obtained for each of the *foxg1* genes (Figure 3B). The patterns obtained with these 5′ and 3′ probes confirmed the results obtained previously with the full-length probes, but the level of background staining was higher when using them (not shown). This is probably due to the insufficient length of these relatively short probes, containing 450–600 nucleotides on average.

The earliest examined stage was the early neurula (stage 17 after Tahara, 1988). The beginning of *foxg1* expression was detected at stage 18. From the midneurula (stage 18) to the late neurula (neural plate closure stage or stage 19), diffuse staining for *foxg1α* and *foxg1γ* gene expression is observed for the neural plate, and foxg1*β* expression is not detected by ISH at these stages (Supplementary Figures S2, S3; Ermakova et al., 2019).

**FIGURE 4:**
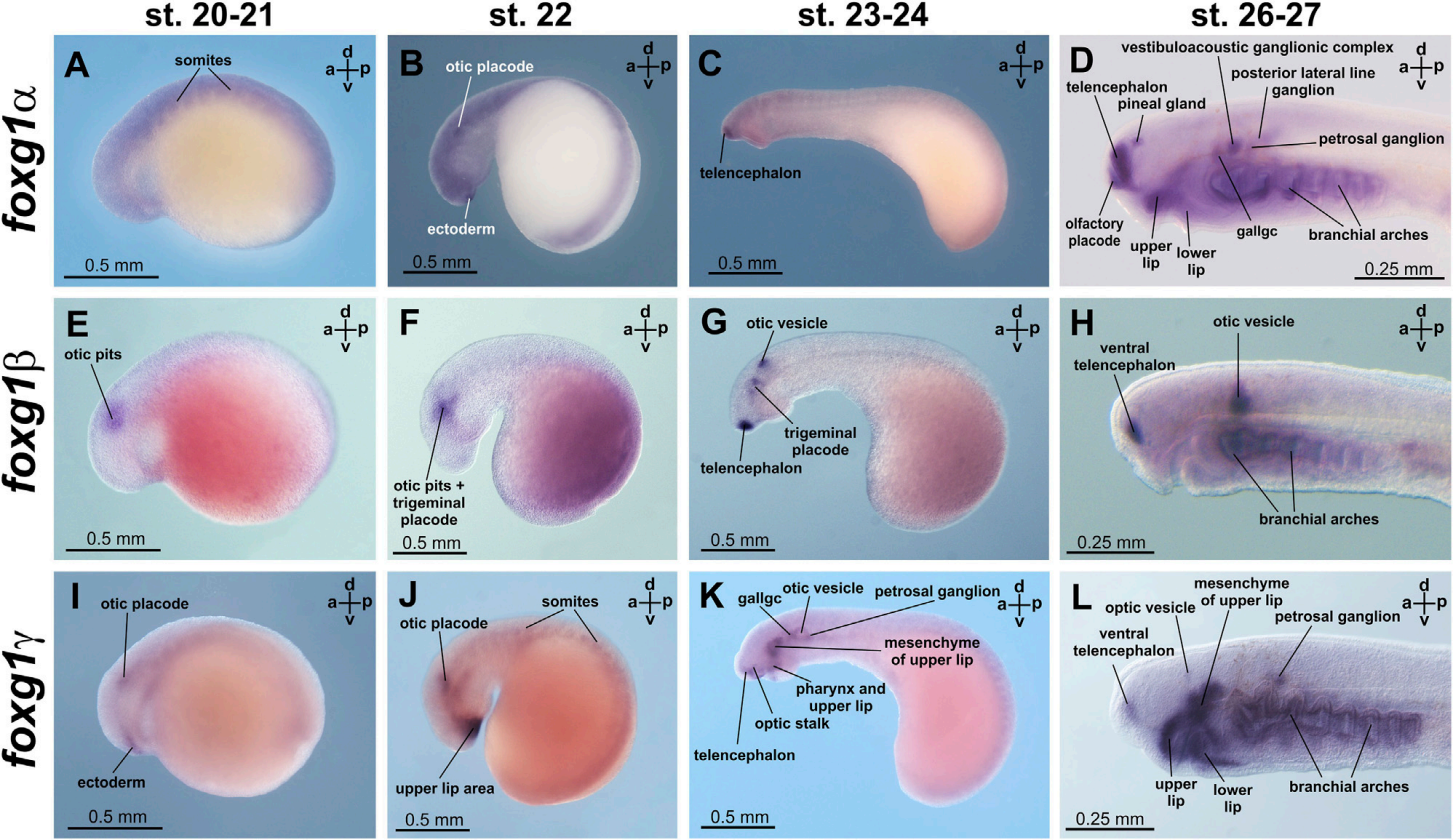
*foxg1* expression in *L. fluviatilis* embryos. gallgc—geniculate/anterior lateral line ganglionic complex. **(A–D)**—*foxg1a* expression, **(E–H)**—*foxg1b* expression, **(I–L)**—*foxg1g* expression.

At stages 20 and 21, diffuse expression of *foxg1α* was detected in neural plate and somite tissues (Figure 4A; Supplementary Figure S2). At stage 22, areas of increased *foxg1α* expression appear in the region of the otic placodes and in a small portion of the ectoderm that lies anterior to the future forebrain (Figure 4B; Supplementary Figure S2).

**FIGURE 5:**
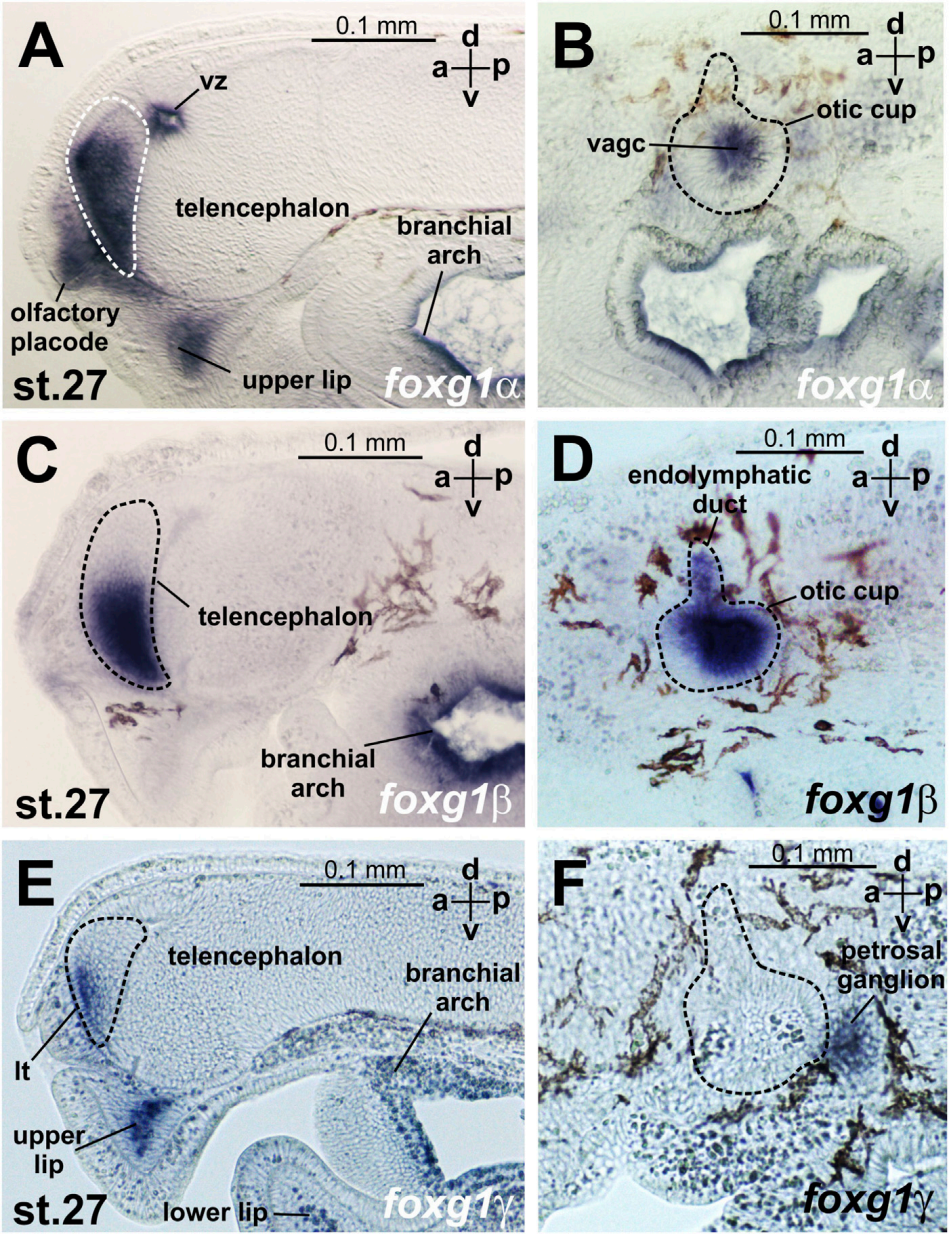
*foxg* expression at sagittal sections head region of *L. fluviatilis* embryos at stage 27. **(A, B)**—*foxg1α,* **(C, D)**—*foxg1β,* **(E, F)**—*foxg1γ*. vz—ventricular zone between habenula, thalamus and telencephalon. vagc—vestibuloacoustic ganglion complex.

At stages 23–24, expression of *foxg1α* appears in the telencephalon (Figure 4C, Supplementary Figure S2). Later, at stages 26–27, *foxg1α* expression is clearly detected throughout the telencephalon, except the small area of the most dorsal part, in the olfactory placode and in the ventricular zone between habenula, thalamus and telencephalon. (Figure 4D; Figure 5A; Supplementary Figure S2). At this stage, zones of *foxg1a* expression are also detected in vestibuloacoustic ganglionic complex (Figure 5B) and other in sensory ganglia: posterior lateral line ganglion, petrosal ganglion, geniculate/anterior lateral line ganglionic complex (Figure 4D). Expression appears in the upper and lower lips and in the branchial arches (Figure 4D; Figure 5B; Supplementary Figure S2).

At stage 30, *foxg1α* expression remains in the forebrain, mouthpart regions, vestibuloacoustical ganglion complex and branchial arches (Supplementary Figures S2I, J).

The expression of *foxg1β* was described by us previously (Ermakova et al., 2019). Briefly, we note that *foxg1β* expression is first detected by ISH at stages 21–22 in the area of the otic placode, trigeminal placode (Figures 4E, F) and later (stage 23–24) in the area of the otic vesicles, trigeminal placode and ventral telencephalon (Figure 4G). At stages 26–27, *foxg1β* is expressed in the ventral telencephalon and otic vesicles (Figure 4H; Figures 5C, D). At stage 30, the expression of *foxg1β* in the ventral telencephalon and otic vesicles continues (Ermakova et al., 2019). It can also be noted that the level of background staining for *foxg1β* is significantly lower than that for *foxg1α*.

*Foxg1γ* at stage 21 is found in the otic placode region (similar to *foxg1β*) and in the thin layer of ectoderm located under the future telencephalon (Figure 4I; Supplementary Figure S3). At stage 22, *foxg1γ* expression in the head ectoderm expands to include the region of the future upper lip, and expression in the otic placode continues (Figure 4J; Supplementary Figure S3).

At stages 23–24, expression of *foxg1γ* begins in the anterior part of the telencephalon and optic stalks. Weak expression of *foxg1γ* is observed in the otic vesicle, geniculate/anterior lateral line ganglionic complex and petrosal ganglion. Expression of *foxg1γ* appears in the dorsal portions of the branchial arches and the mesenchyme of the upper lip (Figure 4K; Supplementary Figure S3).

At stages 26–27, *foxg1γ* is more strongly expressed in the anterior telencephalon with increased expression in the dorsal regions of the branchial arches, continued expression in the upper lip, the mesenchyme of the upper lip and the petrosal ganglion (Figure 4L). Only a weak expression of Foxg1γ was observed in the optic stalks (Supplementary Figure S3H). Expression of Foxg1γ was detected also in lower lip (Figure 5E; Supplementary Figure S3K).

In the telencephalon, as seen in the sagittal section (Figure 5E; Supplementary Figure S3K), *foxg1γ* expression is observed in the narrow, anterior-most portion of the ventral telencephalon and in the lamina terminalis. At this stage, *foxg1γ* expression appears in the anterior part of the optic vesicles (Supplementary Figure S3L).

At stage 30, *foxg1γ* expression remains in the forebrain, petrosal ganglion and mouth region (Figures 5E, F; Supplementary Figure S3M).

Thus, if we try to classify the observed patterns of expression of the three *foxg1* genes of lampreys for their subsequent comparison with homologues of gnathostomes, it can be noted that all three *foxg1* genes of lampreys are expressed in the telencephalon (Figures 5A, C, E), although in different parts of it—*foxg1β* is expressed in the ventral zone of the telencephalon, *foxg1γ* is expressed in the marginal zone of the ventral part of the telencephalon, and the expression of both genes is observed from the 23rd stage. *Foxg1α* is diffusely expressed throughout the telencephalon, except the small area of the most dorsal part, starting from same stages 23–24. Expression in the forebrain and mouth region of all *foxg1* continues until the ammocoete stage (stage 30). All three *foxg1* genes are expressed during the early stages of otic placode formation, although at later stages, only *foxg1β* expression is retained in the otic structures until late stages (Figures 5B, D, F). Only a weak expression of *foxg1γ* was observed in the optic stalks. All three *foxg1* paralogues are expressed in the branchial arches. Additionally, all *foxg1* genes are expressed in the cranial nerve ganglia, but their expression patterns differ. In the upper and lower lips, *foxg1γ* (strongly and with a weak background) and *foxg1α* (weaker) are expressed.

### Expression patterns of *foxg1* genes in sterlet (*A. ruthenus*)

The study of the spatial expression of *foxg1* genes in sterlet was carried out using the ISH method over a series of early stages of development. For the synthesis of ISH probes, cDNAs of the sterlet foxg1 genes were obtained: a probe for the *foxg1a* gene 1100 bp in size (full length of the ORF gene = 1233 bp), a probe for the *foxg1b* gene 900 bp in size (full length of the ORF gene = 1020 bp), and a probe for the *foxg1c* gene 1150 bp in size bp (full length of ORF gene = 1623 bp), containing almost complete cDNAs of the studied genes.

**FIGURE 6:**
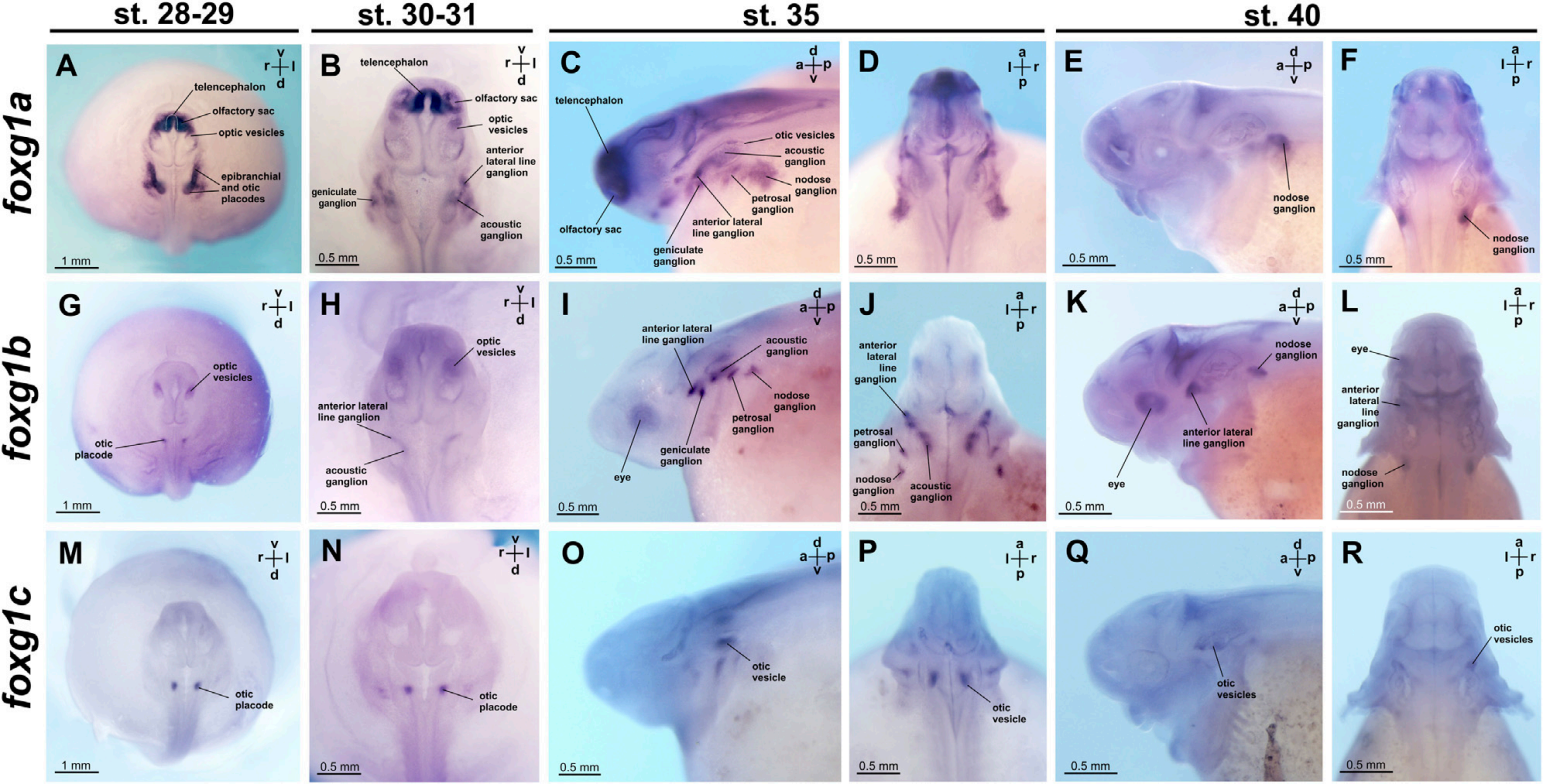
*foxg1* expression in *A. ruthenus* embryos. **(A–F)**—*foxg1a* expression, **(G–L)**—*foxg1b* expression, **(M–R)**—*foxg1c* expression.

Expression of *foxg1a* is already detectable in the telencephalon at the late neurula stage (the earliest of the stages that we studied). At stages 28–29, *foxg1a* is highly expressed in the telencephalon, olfactory sacs and epibranchial/otic placodes. Weak *foxg1a* expression is detected in optic vesicles (Figure 6A; Supplementary Figure S4).

At stages 30–31, high levels of *foxg1a* expression continue to be observed in the telencephalon and olfactory sacs (Figure 6B; Supplementary Figure S4). Expression of *foxg1a* is enhanced in the optic vesicles. *Foxg1a* expression is also detected in geniculate, acoustic and anterior lateral line ganglia.

At stage 35, intense *foxg1a* expression remains in the telencephalon and olfactory sacs, but expression levels decrease in the optic vesicles (Figures 6C, D; Supplementary Figure S4). In the region of the epibranchial placodes, additional *foxg1a* expression appears in the petrosal and nodose ganglia.

At stage 40, *foxg1a* expression remains in the nodose ganglion (Figures 6E, F; Supplementary Figure S4).

The *foxg1b* gene at stages 28–29 is expressed in the optic vesicles and otic placode (Figure 6G; Supplementary Figure S5). At stages 30–31, *foxg1b* continues to be expressed in the optic vesicles and appears in the anterior lateral line ganglion, acoustical ganglion and nodose ganglion (Figure 6H; Supplementary Figure S5). At stage 35, *foxg1b* expression arises in epibranchial ganglia: geniculate and petrosal (Figures 6I, J; Supplementary Figure S5). At stage 40, *foxg1b* expression persists in the optic vesicles, anterior lateral line ganglion and nodose ganglion (Figures 6K, L; Supplementary Figure S5).

Expression of the *foxg1c* gene at the late neurula stage is found only in the otic placodes (Figure 6M; Supplementary Figure S6). Subsequently, at stages 30–31, 35, and 40, *foxg1c* continues to be expressed in the otic vesicles (Figures 6N–R; Supplementary Figure S6).

In summary, only the *foxg1a* gene is expressed in the telencephalon of the sterlet. Moreover, all three *foxg1* genes are expressed in the ear structures and associated ganglia. In the optic vesicles at early stages, only *foxg1b* expression is detected.

Comparing the expression of the three *foxg1* genes in sterlet, it can be noted that the broadest expression pattern is shown by *foxg1a*. It is found in the telencephalon, olfactory sacs, optic vesicles, otic placode, anterior lateral line ganglion and epibranchial ganglia.

The expression pattern of *foxg1b* partially matches that of *foxg1a*; however, unlike *foxg1a*, *foxg1b* is not expressed in the telencephalon and olfactory sacs.

*Foxg1c* expression is found only in the otic placode and subsequently in the optic vesicles.

Based on the analysis of expression features, it can be stated that comparison of *foxg1* gene expression patterns in lamprey and sterlet does not allow us to reliably identify pairs of genes with unique expression features common to both species. In lamprey all three *foxg1* genes are expressed in the telencephalon, whereas in sterlet only *foxg1a* is expressed in this part of the brain. In the otic placodes, during the early stages of otic structure formation in both lamprey and sterlet, expression of all three *foxg1* genes is observed.

*Foxg1γ* is expressed in the optic vesicles of lamprey, *foxg1b* is expressed in sterlet at early stages, and *foxg1a* is expressed at a weaker level. The expression in olfactory structures is similar for *foxg1a* of lamprey and *foxg1a* of sterlet.

Thus, as a result of our comparative analysis of the expression of the *foxg1* genes in river lamprey and sterlet, we did not detect confident pairwise orthology between individual genes, which could support the hypothesis of one or two rounds of general WGD before the separation of the jawless and gnathostome branches in the early evolution of vertebrates.

### Estimation of the relative timing of the second round of *foxg1* gene duplication

Three *foxg1* paralogues are usually present in Agnathans and Gnathostomes lineages. There are deviations from this number—two genes in jawless hagfish, the gnathostome *Callorhinchus* and some reptiles and one gene in birds and mammals. All these deviations are obviously associated with the loss of one or two paralogues from the original three. All detected cases of the presence of four or more genes (sturgeons and teleosts) are associated with independent additional genomic duplications that occurred in these groups at relatively late stages of their evolution.

**FIGURE 7:**
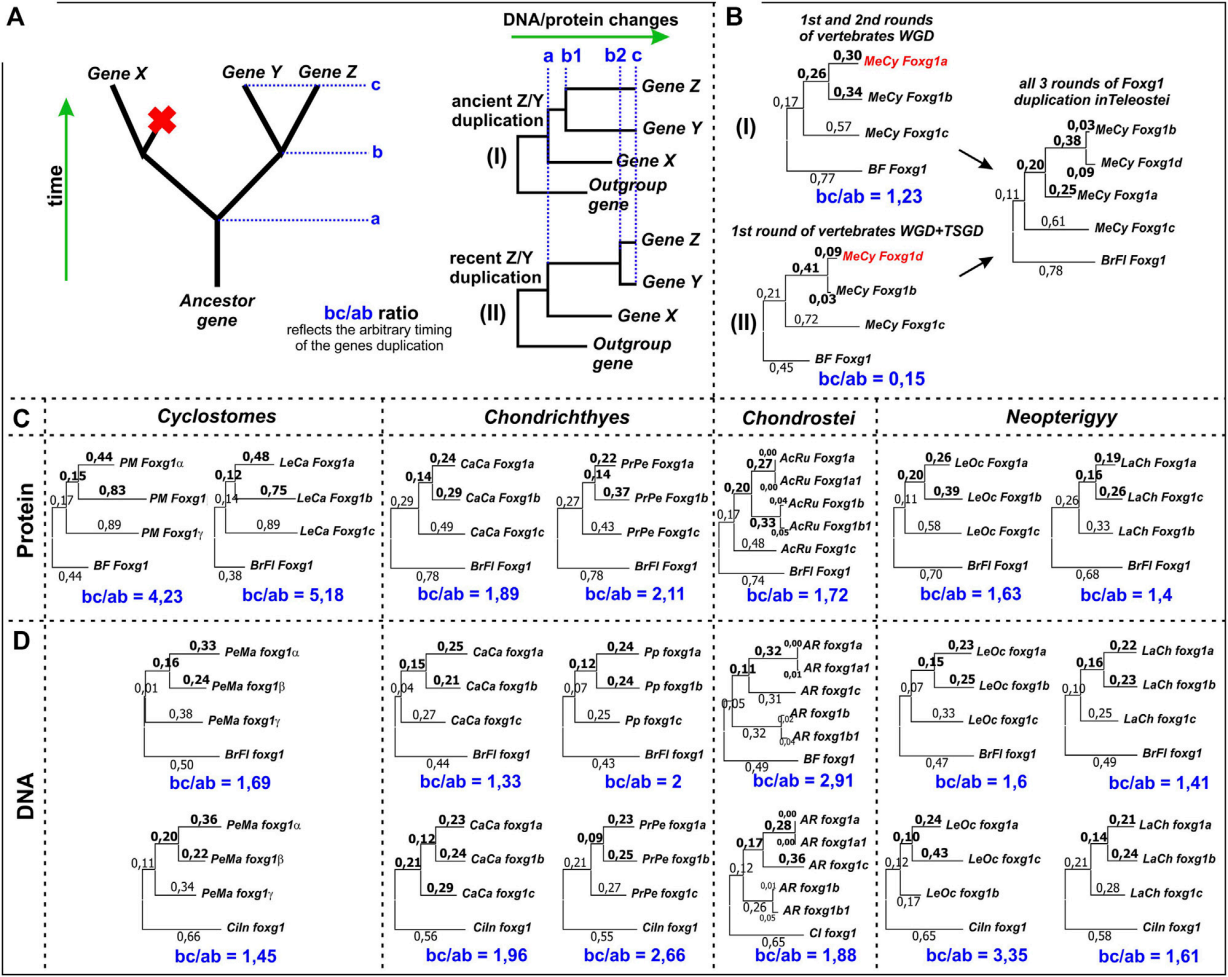
Estimation of the relative timing of the second round of *foxg1* gene duplication in representatives of different groups of vertebrates. **(A)**—the idea of the estimation method used; **(B)**—an example of the application of the estimation method in relation to *foxg1* duplication in Teleostei, whose genome contains 4 paralogues of *foxg1* resulting from 2R early vertebrate (gnathostome) WGD + Teleostei specific WGD (TS-WGD); **(C)**—Estimation of the relative timing of the second rounds of *foxg1* duplication in different vertebrate groups by ML analysis of Foxg1 proteins phylogenetic sequences; **(D)**—The same analysis carried out with *foxg1* nucleotide sequences.

Having sequences of daughter genes/proteins and homologues similar to their ancestral variant (genes/proteins) of the closest relatives (lancelets and tunicates) that did not have genomic duplications, we can try to trace the fates of *foxg1* genes by estimating the time of separation of individual paralogues and compare the times for different groups. The idea and scheme of such an analysis are presented in Figure 7.

By assessing changes in gene/protein sequences, it is possible to correlate the time of appearance of the ancestral gene (a), the time of separation of the two daughter genes (b) and their current state (c). For genes that separated long ago (“ancient” duplications) and recently (“new” duplications), the ratios of the ab and bc segments will differ. This can be seen in the comparison of Foxg1 homologues of teleosts, which have undergone three rounds of duplications - 2 shared with other gnathostomes and a third unique round—the TS-WGD. As follows from the analysis of phylogeny and synteny, *foxg1a*, *foxg1b/d* and *foxg1c* appeared as a result of an ancient common WGD. The first round led to the appearance of two genes, *foxg1a/b/d* and *foxg1c/x*, and the second round led to the appearance of four genes, *foxg1a*, *foxg1b/d*, *foxg1c* and *foxg1x*. The *foxg1x* gene subsequently disappeared. The separation of the *foxg1b* and *foxg1d* genes occurred subsequently—if we assume that they appeared as a result of the TS-WGD, then its timing is estimated at approximately 350–320 million years ago (Pasquier et al., 2016; Du et al., 2020). If the proposed analysis is performed for the *foxg1a*, *foxg1b* and *foxg1c* genes of Indo-Pacific tarpon (*Megalops cyprinoides*), the bc/ab ratio for *foxg1a* and *foxg1b* duplicated in the 2R WDG is 1.23 (Figure 7B (I)). For the *foxg1b* and *foxg1d* gene sets, the ratio is different: 0.15, reflecting the emergence of these genes as a result of the TS-WGD (Figure 7B (II)).

Using this method, one can try to compare the times of the second round of duplication in cyclostomes and gnathostomes.

We assessed the second round of *foxg1* duplication for representatives of different groups of vertebrates. The analysis included assessment of both amino acid (Figure 7C) and nucleotide sequences (Figure 7D). In all cases, the bc/ab ratio was greater than 1, which, by analogy with duplications in Teleostei, corresponds to scenario (I), that is, an ancient duplication. When assessed by DNA, the *foxg1* genes of both the lancelet *Branchiostoma floridae* and the ascidian *Ciona intestinalis* were taken as outgroup genes. In all cases, the estimates were comparable.

In amino acid analysis, the bc/ab ratio in lampreys was approximately 2–3 times higher than that in gnathostomes, which, following the logic underlying the method, indicates a more ancient duplication in agnathans, but probably also reflects the amino acid specificity of lamprey genes (“lamprey dialect”), due to which they are closer to each other in phylogenetic analysis than to orthologues in other groups.

It can also be noted that the analysis revealed almost no difference between the paired *foxg1* paralogues of sturgeons, which appeared as a result of their specific duplication.

Obviously, the results of this analysis do not present rigorous evidence, instead only allowing for a rough assessment of the timing of the duplications in question. Based on the obtained ratios, we can say that *foxg1* duplications apparently occurred in Agnathans and Gnathostomes at the early stages of lineages genotype formation.

## Discussion

In the present article, we describe for the first time the presence of three *foxg1* genes in lampreys, representatives of cyclostomes. The expression of *foxg1* genes in the European river lamprey *L. fluviatilis* was analysed. Additionally, the expression of three *foxg1* genes in the starlet *A. ruthenus*, as a representative sturgeon, one of the oldest groups of gnathostomes available for research today, was described for the first time. Based on phylogenetic, synteny, and expression pattern analyses, we attempted to identify pairs of orthologues (ohnologs) among the *foxg1* genes of cyclostomes and gnathostomes. Such an analysis is of interest in the context of estimating the timing of the second round of WGD at the early stages of vertebrate evolution. The identified features of the primary amino acid sequences, gene environment and expression patterns indicate the common origin of *foxg1* genes in all vertebrates but do not allow us to reliably identify pairs of orthologues in the lineages of cyclostomes and gnathostomes. Thus, the data obtained may correspond with both the “classical” 2R WGD scenario in the common ancestor of vertebrates before the split of the cyclostomes and gnathostomes and the “modern” 1R scenario of an independent secondary duplication in these lineages.

### *Foxg1* genes in lampreys

The presence of one *foxg1* gene in lampreys was described previously (Ermakova et al., 2019; Higuchi et al., 2019). The main paradigm at the time of these works was the presence of one *foxg1* gene in vertebrates, by analogy with amphibians, birds and mammals, in which most of the work on the expression and functional contribution of *foxg1* to brain development was carried out. In addition, one *foxg1* has also been described in the closest relatives of vertebrates—lancelets and tunicates. Productive genome screening was further complicated for a long time by the incompleteness of the cyclostome genomic sequences available. Our present work shows that three *foxg1* paralogues are present in both lampreys and some evolutionarily basal groups of gnathostomes. The analysis carried out in this article allows us to conclude that previous descriptions of *foxg1* in lampreys referred to different paralogues: in the article of Higuchi et al. (2019), *foxg1α* was described, while in the article of Ermakova et al. (2019), it was *foxg1β*.

In the article by Higuchi et al. (2019), the *foxg1α* pattern was shown in the context of the development of inner ear structures in cyclostomes (see Higuchi et al., 2019, Extended Data Figure 7). Expression was studied in the Arctic lamprey *L. camtschaticum* at stage 25, while in our present work, the ISH pattern study was carried out in the river lamprey *L. fluviatilis* at a series of early stages. The probe of Higuchi et al. (2019), according to the given description, corresponded to the 5′ probe of our study; that is, it included the 5′ region upstream of the highly conserved FB domain (https://www.ncbi.nlm.nih.gov/protein/BBG56415.1/). Common features of the observed *foxg1α* pattern in both studies include expression throughout the telencephalon (while the other genes, *foxg1β* and *foxg1γ,* are expressed only in the ventral part of the telencephalon) and a strong background signal (see Higuchi et al., 2019, Extended Data Figure 7B).

Ermakova et al. (2019) previously described the *foxg1β* gene in the European river lamprey *L. fluviatilis*.

In the present work, we described for the first time all three *foxg1* genes in lampreys, investigated their phylogenetic relationships with genes of other vertebrates, and described their expression features at a series of early stages of river lamprey development. The data obtained complement and systematize our knowledge about *foxg1* genes in cyclostomes, as one of the evolutionarily ancient lineages of vertebrates.

### *Foxg1* genes in basal gnathostomes

Three *foxg1* genes in sharks were described by Hara and colleagues (Hara et al., 2018). The paper presented *de novo* whole-genome assemblies of brown-banded bamboo shark and cloudy catshark and an improved assembly of the whale shark genome. The *FoxG3* gene (which we propose to be considered as *foxg1b*, with arguments given above in the section on phylogenetic analysis) has attracted attention in the framework of genomic analysis because it disappeared in tetrapods. Studies of the expression patterns of *foxg* genes in catshark (*S*. *torazame*) at stages 24 and 27 were also presented (see Hara et al., 2018; Figure 4; Supplementary Figure S6). *FoxG1* (*foxg1a*) is expressed in the telencephalon, ocular structures, acoustico-facial ganglionic complex and vagal ganglion. At stage 27, the onset of expression in the branchial arch primordia was noted (see Hara et al., 2018, Supplementary Figure S6C). The *FoxG3* gene (*foxg1b*) at stage 23 was expressed in an anterodorsal part of the retina, in the acoustico-facial ganglionic complex, and at a weak level in the vagal ganglion and ventral end of presumptive branchial arches (see Hara et al., 2018; Figure 4F). At stage 27, expression in the vagal ganglion increases and disappears in the branchial arches. The *FoxG2* gene (*foxg1c*) was expressed in the acoustico-facial ganglionic complex and the vagal ganglion (see Hara et al., 2018; Figure 4E). Thus, shark *foxg1* genes exhibit spatial subfunctionalization. Along with common expression areas (acoustico-facial ganglionic complex and the vagal ganglion), each of the *foxg1* genes also has unique features. For example, *foxg1a* shows expression in the telencephalon, otic vesicle, ventral part of the ocular structures (at stage 23) and branchial arches (at stage 27), and *foxg1b* is expressed in an anterodorsal part of the retina.

In the present work, we conducted a study of the expression patterns of three *foxg1* paralogues in another evolutionarily ancient group of gnathostomes, sturgeons, taking the sterlet *A. ruthenus* as an example. The sterlet genome has been sequenced and is publicly available (Du et al., 2020, https://www.ncbi.nlm.nih.gov/datasets/genome/GCF_010645085.2/). A feature of sturgeon genomes is that polyploidy arose as a result of duplications in the ancestors of the group. Due to this feature, paralogues of individual genes can be present in more copies than in other groups of vertebrates. At the same time, our phylogenetic and synteny analyses showed the presence of three basic *foxg1* paralogues in sturgeons orthologous to the genes of other gnathostomes. The expression patterns of sterlet *foxg1* genes show common features with the *foxg1* genes of sharks. The technical advantage of sterlet in spatial expression analysis is external fertilization and the ability to culture eggs in fresh water in Petri dishes, similar to lamprey eggs or eggs of the standard laboratory model *Xenopus*. In sharks, embryos develop inside the dense shell of the egg and are inaccessible for external observations (Musa et al., 2018). Along with the small number of eggs in each clutch and the difficulties of maintenance, this creates difficulties in obtaining a sufficient number of shark embryos for the needed stages of development.

The genomic signature of sturgeons is lineage-specific genome duplication(s) in addition to the early 2R WGD common to gnathostomes. *Acipenser* and *Polyodon* represent two early divergent sturgeon lineages. It is debatable whether the sturgeon duplication occurred in their common ancestor before the split of these lineages, or whether separate duplications occurred independently in *Acipenser* and *Polyodon*. Clustering of sturgeon paralogs of Foxg1a, where paralogs from one species (e.g., *Acipenser*) are closer to each other than their corresponding orthologs from another species, argues for an independent duplication of these genes in the two sturgeon genera (Figure 1, red dotted frame). At the same time, the clustering of Foxg1b, where *Acipenser* and *Polyodon* orthologs are closer to each other than to their paralogs, suggests a duplication of their common ancestor. The Foxg1a sturgeon emergence scenario corresponds to the previously described Lineage-specific Ohnologue Resolution (LOR) model, while the Foxg1b clustering pattern corresponds to the Ancestral Ohnologue Resolution (AOR) model (Robertson et al., 2017). Taken together, the combination of both models of rediploidization in one genome corresponds well to the model of common WGD followed by asynchronous rediploidization described for sturgeons (Redmond et al., 2023). Significantly, there is also evidence for a *foxg1b* duplication at the level of the common sturgeon ancestor prior to the split of the *Acipenser* and *Polyodon* lineages, which may provide additional evidence for an ancestral sturgeon WGD.

According to our data, the *foxg1a* of sterlet is expressed in the telencephalon, olfactory sacs, optic vesicles and geniculate, acoustic, anterior lateral line, petrosal and nodose ganglia. *Foxg1b* is expressed in olfactory sacs, otic placodes and vesicles, and the same ganglia as *foxg1a*. *Foxg1c* is expressed in otic placodes and vesicles.

When comparing the *foxg1* patterns of sterlet and catshark, common features of the expression of orthologous genes can be noted. *Foxg1a* has the broadest expression pattern in both species, being found in the telencephalon, optic and otic structures, and epibranchial ganglion complex. *Foxg1b* also shows expression in the optic vesicles and ganglion complexes. *Foxg1c* in both species shows the most restricted expression pattern in the otic structures and ganglion complexes (in sharks).

Thus, within the gnathostome lineage, the orthology of the three paralogues of the *foxg1* genes is convincingly traced according to all tests performed - analysis of phylogenetic relationships, local genomic synteny and expression patterns. At the same time, pronounced spatiotemporal subfunctionalization was not observed in the three *foxg1* genes. The expression pattern of *foxg1a* mainly overlaps with and includes the expression patterns of *foxg1b* and *foxg1c*, which may reflect the preconditions for their disappearance in evolutionarily younger groups.

In the context of the second round of WGD in vertebrates, it is of interest to identify pairs of orthologues in representatives of cyclostomes and gnathostomes. It was not possible to do this based on the phylogenetic and local genomic synteny analyses. In the phylogenetic analysis, two of the three jawless genes clustered more strongly with each other than with the gnathostome genes. Regarding *foxg1* synteny, lampreys have common neighbours with all gnathostome genes, which also does not allow us to identify their orthologues. Analysis of the expression pattern in this case may be necessary to clarify the situation.

To formalize the comparison and correlation of gene expression features of lampreys with sterlet and catshark, we compiled expression description tables (Tables 1, 2).

**TABLE 1:** Comparison of the expression of *foxg1* in certain developing embryonic structures of *L. fluviatilis* and *A. ruthenus*.

	*Ar_foxg1a*	*Ar_foxg1b*	*Ar_foxg1c*
*Lf_foxg1* *α*	Telencephalon	Otic placode	Otic placode
	Otic placode		
	Olfactory placode		
*Lf_foxg1* *β*	Ventral telencephalon	Otic placode	Otic placode
	Otic placode/vesicle		
*Lf_foxg1* *γ*	Ventral telencephalon	Otic placode	Otic placode
	Otic placode	Optic vesicle	
	Optic vesicle	Epibranchial ganglia	
	Epibranchial ganglia		

**TABLE 2:** Comparison of the expression of *foxg1* in certain developing embryonic structures of *L. fluviatilis* and *S. torazame* (after Hara et al., 2018).

	*St_foxg1a*	*St_foxg1b*	*St_foxg1c*
*Lf_foxg1* *α*	Telencephalon		
	Otic placode		
	Olfactory placode		
*Lf_foxg1* *β*	Ventral telencephalon	Branchial arches (weak)	
	Otic placodes		
	Branchial arches		
*Lf_foxg1* *γ*	Ventral telencephalon	Optic vesicle	Epibranchial ganglia
	Otic placodes	Epibranchial ganglia	
	Optic vesicle (early)		
	Epibranchial ganglia		

The comparison shows that, similar to the result from the analysis of local genomic synteny, the *foxg1* genes of lampreys and gnathostomes exhibit multiple intersections, which make it difficult to identify pairs of orthologues. Thus, in lampreys, all three *foxg1* genes are expressed in the telencephalic region, while in gnathostomes, only *foxg1a* is expressed there. All three *foxg1* genes are expressed in the otic structures of lampreys and sterlets. In the optic vesicles of lampreys, only *foxg1γ* is expressed, while in sharks and sturgeons, *foxg1a* and *foxg1b* are expressed. Such multiple intersections of expression patterns, on the one hand, confirm the similarity of regulatory elements and the common origin of *foxg1* genes as a result of WGD, but they do not provide convincing evidence in favour of one or two common rounds of duplication in ancestral vertebrates to clarify the separation of the cyclostomes and gnathostomes.

Our assessment of the relative timing of the second round of *foxg1* duplications in the lineages of lamprey and gnathostomes shows, that in both cases, the evolutionary events took place at the early stages of the formation of the genotype of both lineages were relatively ancient. These rounds of duplications predated the TS-WGD, which is estimated to have approximately 320–350 million years ago (Jaillon et al., 2004). Taking into account the fact that in the scenario of independent second rounds of polyploidization in cyclostomes and gnathostomes, the antiquity of these events in two lineages is estimated similarly—approximately 450–460 million years ago (Marlétaz et al., 2023), our data on the three paralogues of *foxg1* in lampreys and basal gnathostomes appear consistent with both the 1R and 2R scenarios of WGD in vertebrates.

## Materials and methods

### Animals and samples preparation

All animal experiments were performed in accordance with guidelines approved by the Shemyakin-Ovchinnikov Institute of Bioorganic Chemistry (Moscow, Russia) Animal Committee and handled in accordance with the 1986 Animals (Scientific Procedures) Act and Helsinki Declaration.

Adult mature individuals of the European river lamprey *L. fluviatilis* were caught in the Leningrad district. Developing embryos were obtained through artificial insemination in laboratory. Lamprey stages were determined according to Tahara (Tahara, 1988).

The *A. ruthenus* eggs and embryos were obtained and collected in Tver district, Konakovo, Russia. The embryos of *A. ruthenus* were staged in accordance with in accordance with Ginsburg and Dettlaf (1975) and Shmalgauzen (1975).

For ISH, embryos were fixed in MEMFA solution (3.7% formaldehyde, 100 mM MOPS, 2 mM EGTA, 1 mM MgSO4), dehydrated in methanol and kept at −20°C.

*L. fluviatilis* total RNA samples of set of stages were obtained from lysed embryos (50 embryos for probe) by purification with the Analytic Jena innuPREP RNA Mini Kit 2.0 (Berlin, Germany).

### Analyses of phylogeny and synteny

The search for homologs was carried out in Blastn (https://blast.ncbi.nlm.nih.gov/Blast.cgi?PROGRAM=blastn&PAGE_TYPE=BlastSearch&BLAST_SPEC=&LINK_LOC=blasttab&LAST_PAGE=blastn) and tBlastn (https://blast.ncbi.nlm.nih.gov/Blast.cgi?PROGRAM = tblastn&PAGE_TYPE = BlastSearch&BLAST_SPEC = &LINK_LOC = blasttab&LAST_PAGE = blastn) sections. We checked available Nucleotide collections (nr/nt) and wholegenome shotgun contigs (wgs).

Multiple alignment was performed by ClustalW algorhythm in the MEGA11 program.

Phylogenetic analyses of FoxG1 protein sequences of vertebrates were performed via the Maximum Likehood (ML) and Neighbor-Joining (NJ) methods using the MEGA11 program (Tamura et al., 2021).

The choosing of optimal model was made in MEGA11. The results are present in Supplementary Table S1.

In ML method JTT matrix-based model (Jones et al., 1992) with frequencies and Gamma distribution was used. The percentage of trees in which the associated taxa clustered together in the bootstrap test (500 replicates) is shown next to the branches (Felsenstein, 1985). The tree is drawn to scale, with branch lengths measured in the number of substitutions per site. This analysis involved 80 amino acid sequences. There were a total of 814 positions in the final dataset.

In NJ method analysis (Saitou and Nei, 1987) the optimal tree is shown. The percentage of replicate trees in which the associated taxa clustered together in the bootstrap test (500 replicates) are shown next to the branches (Felsenstein, 1985). This analysis involved 80 amino acid sequences. All ambiguous positions were removed for each sequence pair (pairwise deletion option). There were a total of 814 positions in the final dataset.

The list of the analyzed Foxg1 sequences is attached in Supplementary Information.

Synteny analysis and search for neighboring genes were also carried out on the NCBI website (https://www.ncbi.nlm.nih.gov/).

The phylogenetic analysis presented in Figure 7 was performed using the same sequences and algorhythms (ClustalW multiple alignment and ML trees) as Figure 1.

### *FoxG1* cDNA obtaining, RT-PCR, ISH

cDNAs of *L. fluviatilis foxg1* genes were obtained by nested PCR with following pairs of primers (synthesed by Evrogen, Moscow):

*Lf_foxg1α_ LC_full_1_frw;* AAT​ACA​GCA​GCG​TGG​ACA​TGC​TG; 0.04

*Lf_foxg1α_ LC_full_2_frw;* AAT​ATG​CTG​GAC​ATG​GGC​GAT​CA; 0.04

*Lf_foxg1α_ LC_full_1_rev;* AAT​TCA​CCA​CCA​CCA​CCG​TCA​GTG; 0.04

*Lf_foxg1α_ LC_full_2_rev;* AAT​TCA​GTG​TAA​GAG​ACT​GTT; 0.04

*Lf_foxg1γ_ LC_full_1_frw;* AAT​GAC​CAG​GGA​GGG​GGA​TGC​C; 0.04

*Lf_foxg1γ_ LC_full_2_frw;* AAT​ATG​CCG​GAC​ATG​GCA​GAC​C; 0.04

*Lf_foxg1γ_ LC_full_1_rev;* AAT​CTG​GGA​TAT​CTT​CCT​CAG​TG; 0.04

*Lf_foxg1γ_ LC_full_2_rev;* AAT​TCA​GTG​TCC​GAA​ATA​AGC​C; 0.04

*L. fluviatilis Foxg1β* ISH probe was obtained as described in Ermakova et al. (2019).

For 5′ and 3’ probes shown at Figure 4B additional primers were used:

*Lf_foxg1α_in situ1_rev* CCA​TCA​TGA​TGA​GCG​CGT​TG

*Lf_foxg1α_in situ2_frw* GCT​TTC​CGC​CGC​GGC​CCT​CT

*Lf_foxg1β_in situ1*_rev GAA​CGG​AGG​CTT​CTC​GTA​CT

*Lf_foxg1β_in situ2*_frw GCT​GTA​CTG​GCC​CGT​ATC​GC

*Lf_foxg1γ_in situ2*_frw CGGCGCTCCGCGGTGTCT

*Lf_foxg1γ_in situ1*_rev ATG​AGC​GCG​TTG​TAG​CTG​AA

cDNAs of *A. ruthenus foxg1* genes were obtained by nested PCR with following pairs of primers:

*AR_foxg1a_in situ_Frw1*; AAA​CAG​CCT​GGT​GCC​TGA​AGC

*AR_foxg1a_in situ_Frw2*; TGA​CAA​CCA​CCA​CAG​ATC​AG;

*AR_foxg1a_in situ_Rev1*; TAG​TGT​ATA​AGA​GGG​TTT​GA

*AR_foxg1a_in situ_Rev2*; CTG​ACT​GTG​ATG​TGG​GAA​GT

*AR_foxg1b_in situ_Frw1*; GGA​TCA​GAA​AGA​GCC​GAC​A

*AR_foxg1b_in situ_Frw2*; GAG​CCT​GCT​GTT​TCC​TTC​TAA

*AR_foxg1b_in situ_Rev1*; TCA​GTT​TAA​AAA​CGA​ACT​AG

*AR_foxg1b_in situ_Rev2*; AAC​CCT​GTT​TTG​ATG​CGA​CA

*AR_foxg1c_in situ_Frw1*; GGA​TTG​TCC​GCG​CGT​CTT​CA

*AR_foxg1c_in situ_Frw2*; GCG​CAC​GCT​GAT​ACT​TCC​AG

*AR_foxg1c_in situ_Rev1*; CTC​TGC​CGC​TGG​TGT​CCA​GG

*AR_foxg1c_in situ_Rev3*; GCT​AAG​TTC​TAC​CTC​AGC​AG

In the first round of PCR (30 cycles), primers Frw1 and Rev1 were used. The resulting PCR product was purified and used as a template in the next round of PCR (20 cycles) with primers Frw2 (which contains Kozak sequence and start ATG) and Rev2. PCR was performed with Encyclo polymerase Evrogen kit (www.evrogen.ru, Moscow).

The resulting cDNA fragments were cloned into the pAL2-T vector (Evrogen, Moscow) and cDNA inserts of 3 clones of each *Noggin* were sequenced. To obtain mRNA for injection, *Noggin2* and *Noggin4* cDNAs were recloned into the pCS2 vector. mRNA synthesis was carried out by SP6 mMessage mMachine kit (Thermo Fisher Scientific, Waltham, Massachusetts).

ISH was carried out according to the protocol reported by Bayramov et al. (2011) and Ermakova et al. (2020).

Photography was carried out using a Leica M205 stereo microscope.

For qRT-PCR analysis first strand samples were obtained using total RNA samples collected in three repetitions. The concentration of the extracted RNA was measured with a Qubit^®^ fluorometer (Invitrogen), while RNA integrity was checked visually via gel electrophoresis.

Two independent pairs of primers for each of lamprey *foxg1* genes were used to exclude unspecific signals. For the arbitrary unit in Figure 4A, we take the expression level at the earliest investigated stage—the early blastula. First strand synthesis, qPCR and data analysis were performed according to Ermakova et al. (2020).

Three independent pairs of primers were used for each of *foxg1* genes to exclude unspecific signals. The following pairs of primers designed by Primer-Blast tool on the base of full-length *L. fluviatilis* sequences were used:

*foxg1a_ Lf_RT_2_frw;* GAAGCCAGCGACGGGAG; 0.04

*foxg1a_ Lf_RT_2_rev;* GTT​GGG​ACA​GCT​ACA​CCG​AT; 0.04

*foxg1a_ Lf_RT_3_frw;* CAC​TCT​GGC​GGG​TTG​ATT​CC; 0.04

*foxg1a_ Lf_RT_3_rev;* GTT​GGC​TGA​ATG​TCC​CGT​CT; 0.04

*foxg1a_ Lf_RT_5_frw;* TCCGGAGGCGGGGAG; 0.04

*foxg1a_ Lf_RT_5_rev;* TCCGGGCTACGCGGC; 0.04

*foxg1b_ Lf_RT_1_frw;* GAGGGATGCGACGAGGC; 0.04

*foxg1b_ Lf_RT_1_rev;* TAG​CTG​AAG​GGC​GGT​TTC​T; 0.04

*foxg1b_ Lf_RT_2_frw;* ATG​GGC​TTA​GAG​GCT​TTC​GG; 0.04

*foxg1b_ Lf_RT_2_rev;* ACT​TGA​CTT​TGC​TGC​TGA​GGT; 0.04

*foxg1b_ Lf_RT_3_frw;* GCT​CTG​ACT​GGC​CTA​CAC​G; 0.04

*foxg1b_ Lf_RT_3_rev;* GTA​GAA​GGC​GGA​GAG​TGC​TG; 0.04

*foxg1γ_ Lf_RT_1_frw;* TCT​TCC​TTC​TTC​AGC​ATC​GCC; 0.04

*foxg1γ_ Lf_RT_1_rev;* CAC​GAC​AGT​GAG​TCC​GGG​T; 0.04

*foxg1γ_ Lf_RT_2_frw;* AGG​CTT​CCT​CTT​CCT​TCT​TCA​G; 0.04

*foxg1γ_ Lf_RT_2_rev;* GAG​TCC​GGG​TCG​GGA​GAT; 0.04

*foxg1γ_ Lf_RT_3_frw;* TGC​TGC​CTC​TGC​ATT​CTC​AT; 0.04

*foxg1γ_ Lf_RT_3_rev;* GAC​CTG​CTG​CGT​GGT​TAC​T; 0.04

## Data Availability

The original contributions presented in the study are included in the article/Supplementary Material, further inquiries can be directed to the corresponding authors.

## Supplementary material

SUPPLEMENTARY FIGURE S1 Unrooted ML tree of *Foxg1* proteins of cyclostomes and cyclostomes with gnathostomes having three *foxg1* paralogues.

SUPPLEMENTARY FIGURE S2
*foxg1α* expression in *L. fluviatilis* embryos. gallgc—geniculate/anterior lateral line ganglionic complex.

SUPPLEMENTARY FIGURE S3
*foxg1γ* expression in *L. fluviatilis* embryos. lt—lamina terminalis, gallgc—geniculate/anterior lateral line ganglionic complex.

SUPPLEMENTARY FIGURE S4
*foxg1a* expression in *A. ruthenus* embryos.

SUPPLEMENTARY FIGURE S5
*foxg1b* expression in *A. ruthenus* embryos.

SUPPLEMENTARY FIGURE S6
*foxg1c* expression in *A. ruthenus* embryos.

SUPPLEMENTARY FIGURE S7 ML phylogenetic trees of *Foxg1* proteins of vertebrates constructed by Dayhoff w/freq. model (A; Schwarz and Dayhoff, 1979) and Le and Gascuel model (Le and Gascuel, 2008). Lamprey *Foxg1* are violet, sturgeon *Foxg1* are red.

SUPPLEMENTARY TABLE S1 Results of the models testing for phylogenetic analysis.
