# Supplementary figures and images for: Three foxg1 paralogues in lampreys and gnathostomes—brothers or cousins?
Source: Front Cell Dev Biol. 2024 Jan 2;11:1321317. doi: 10.3389/fcell.2023.1321317 (PMC10789856; doi:10.3389/fcell.2023.1321317)

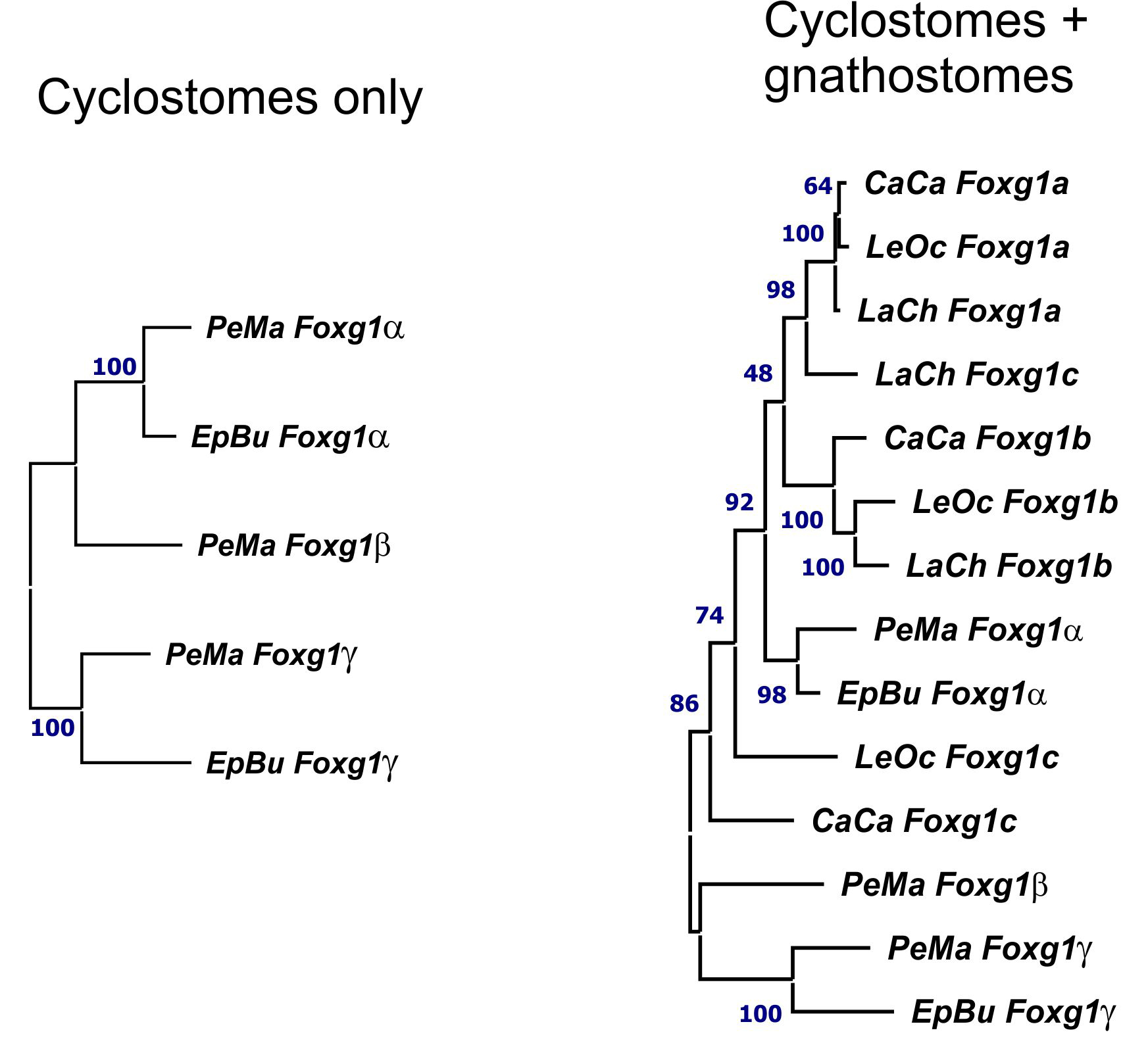

Supplement: Supplementary file 1 [file Presentation1.zip › Image 1.jpg]

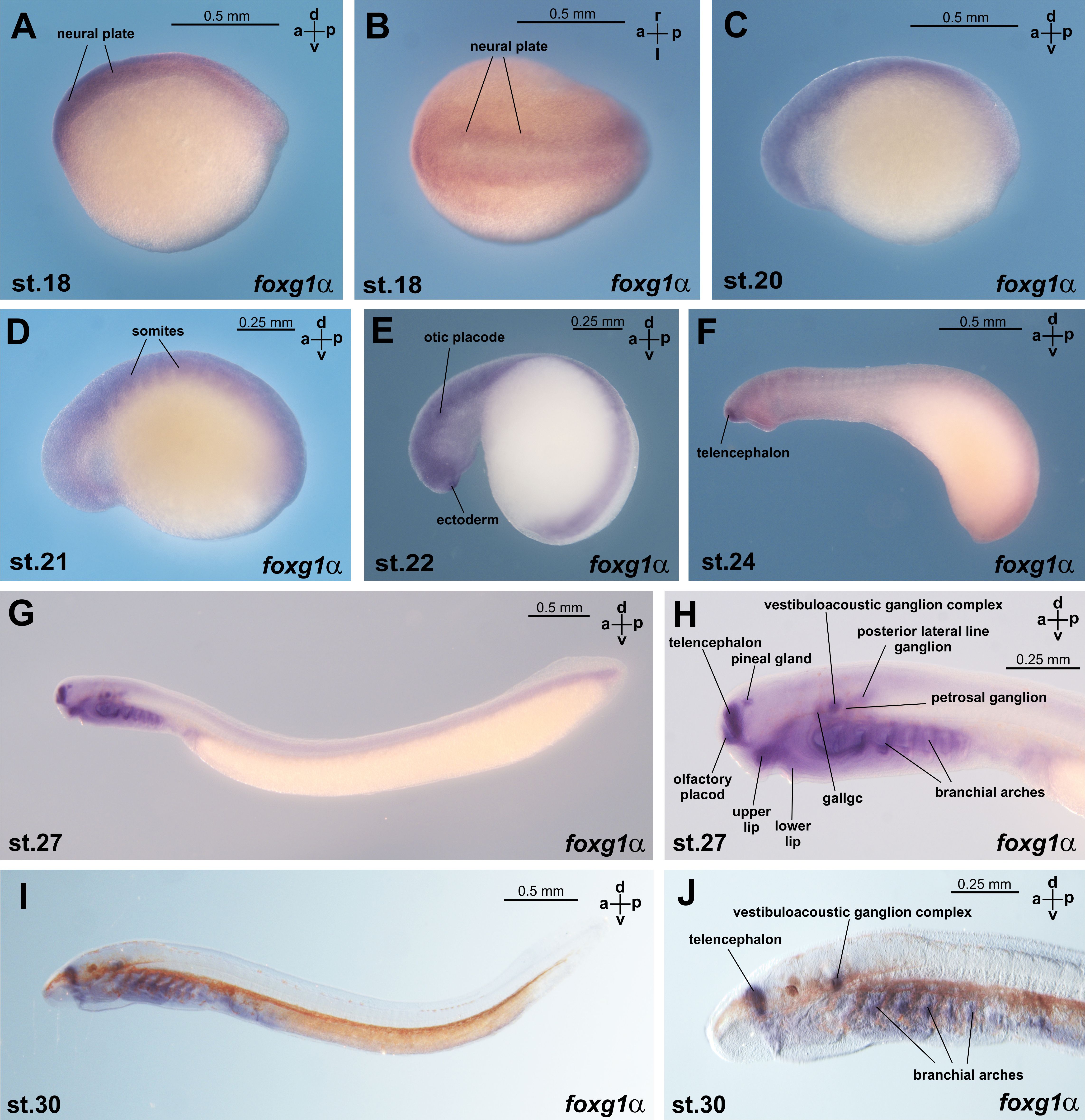

Supplement: Supplementary file 1 [file Presentation1.zip › Image 2.jpg]

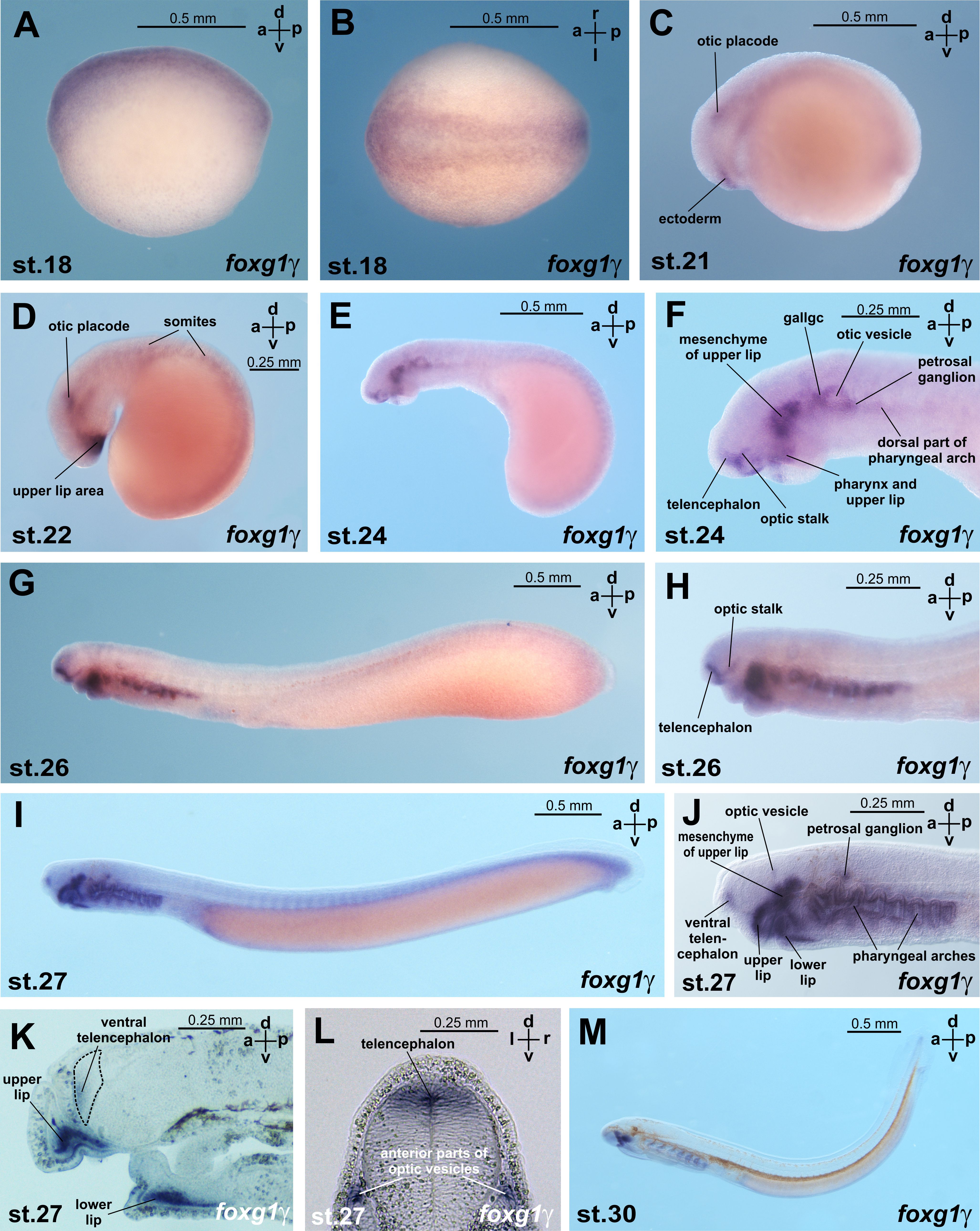

Supplement: Supplementary file 1 [file Presentation1.zip › Image 3.jpg]

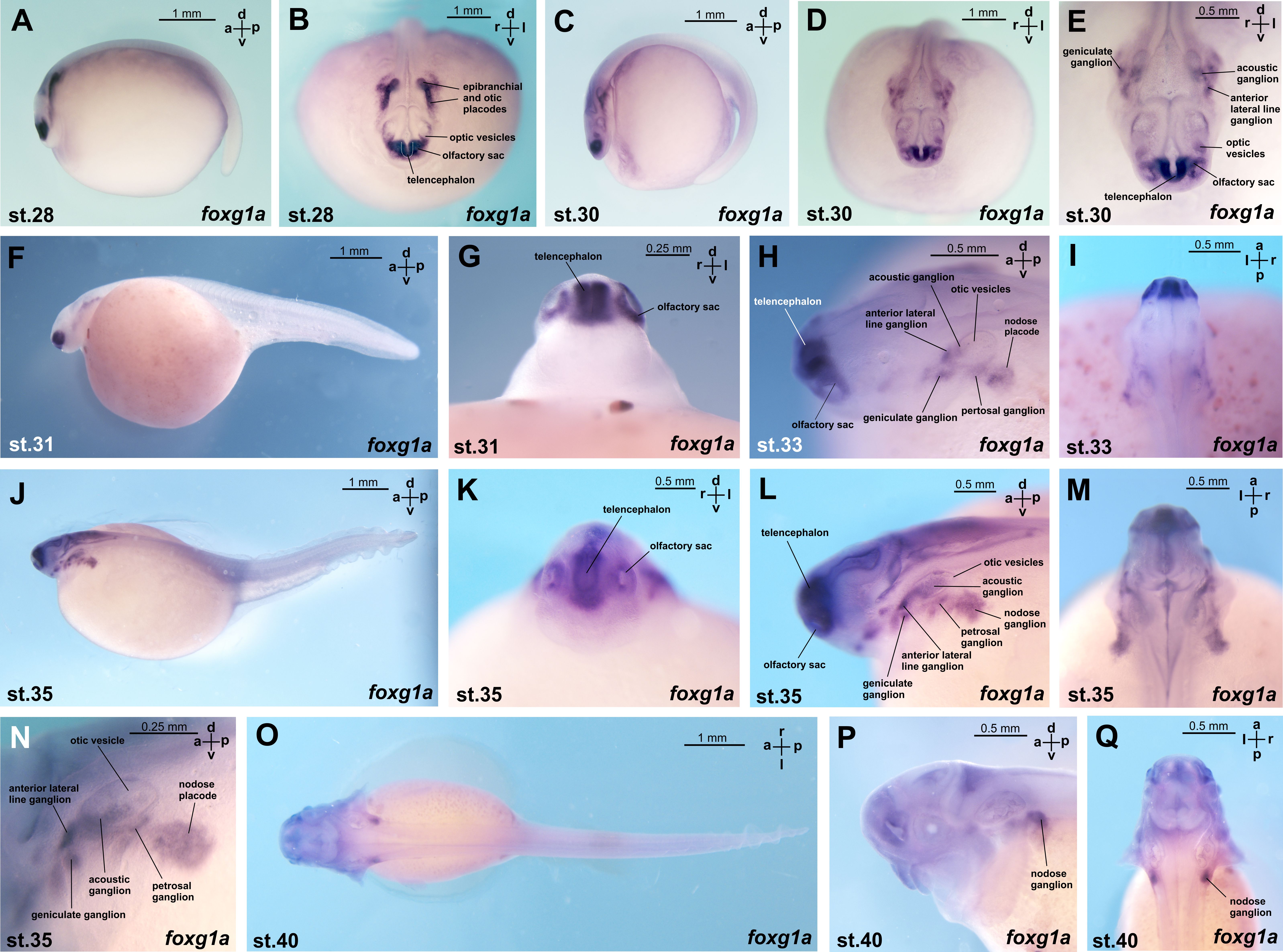

Supplement: Supplementary file 1 [file Presentation1.zip › Image 4.jpg]

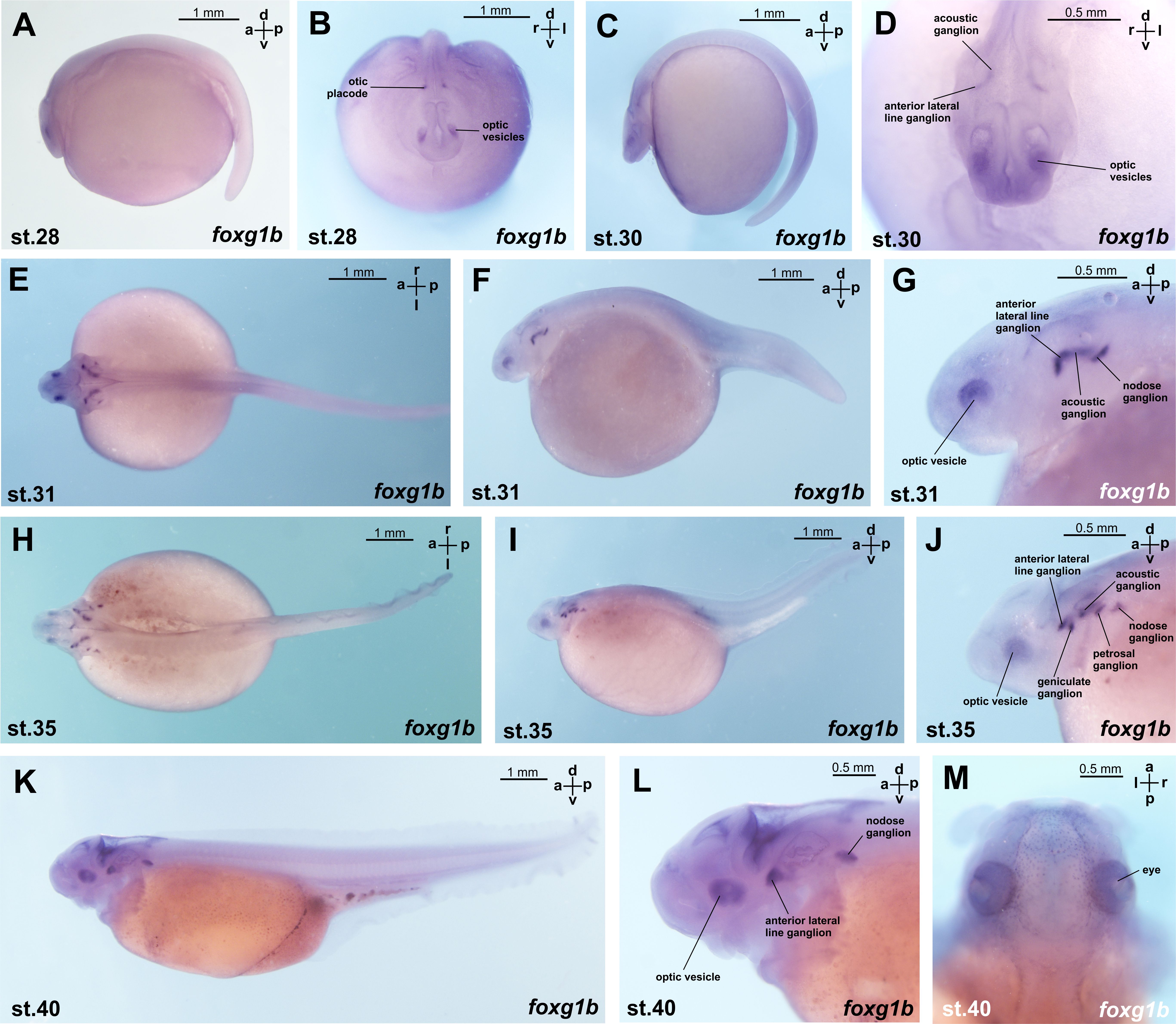

Supplement: Supplementary file 1 [file Presentation1.zip › Image 5.jpg]

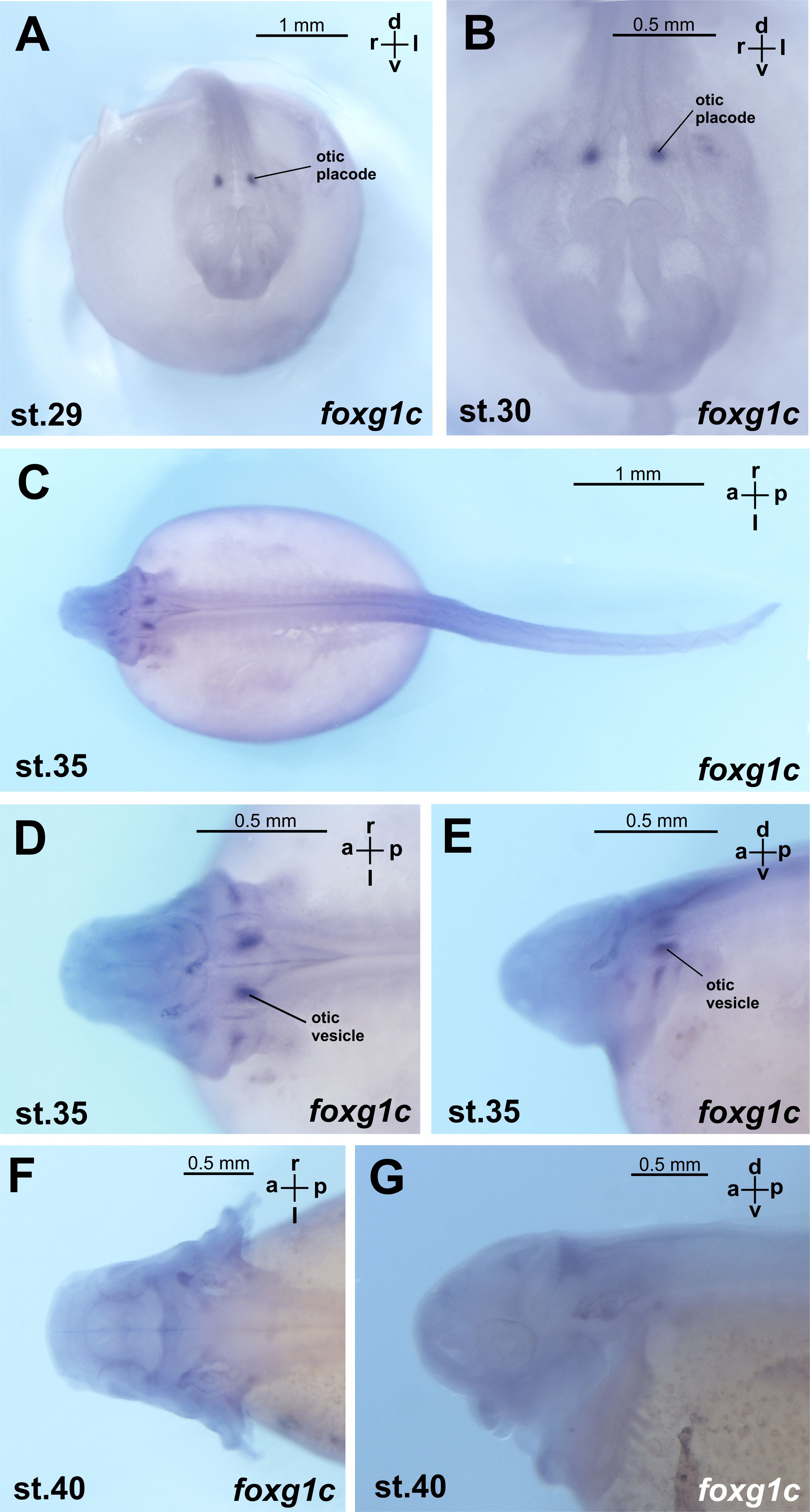

Supplement: Supplementary file 1 [file Presentation1.zip › Image 6.jpg]

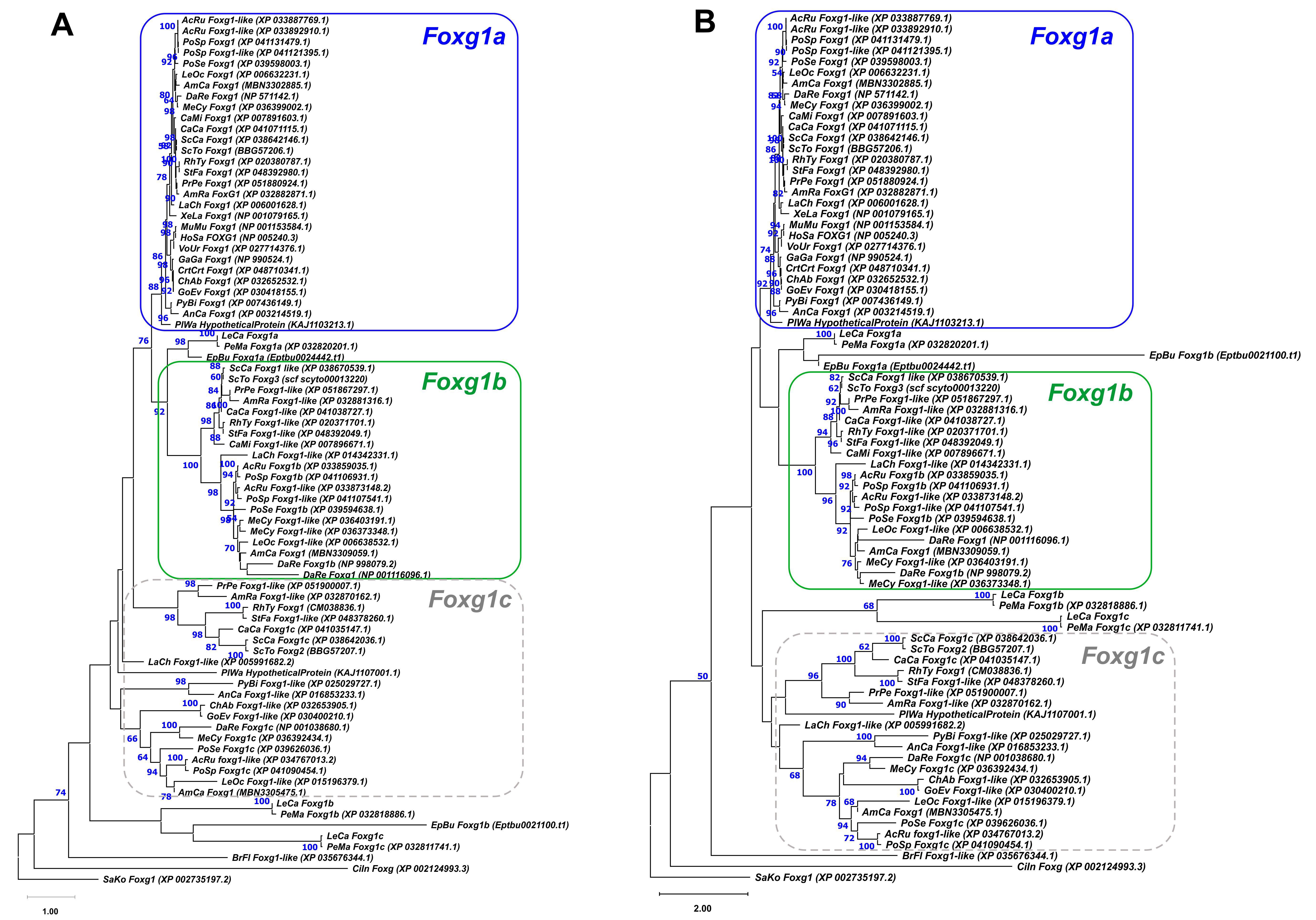

Supplement: Supplementary file 1 [file Presentation1.zip › Image 7.jpg]
