# Supplementary material for: Three foxg1 paralogues in lampreys and gnathostomes—brothers or cousins?
Source: Front Cell Dev Biol. 2024 Jan 2;11:1321317. doi: 10.3389/fcell.2023.1321317 (PMC10789856; doi:10.3389/fcell.2023.1321317)
Supplement: Supplementary file 2 [file DataSheet1.docx]

***Supplementary file***

**Foxg1 sequences included in phylogenetic analysis.**

>BrFl_Foxg1-like (XP_035676344.1)

MVRTEDRSPECPSNINDCPFSIRRMLSQPLHTAELPTVSLTAGHAPAPVTCRETNGDHSHHNKAVNEHEL

GKDGKDAPDSKVDTDVPKDSEQEDFKKEKDDKEEGKKHEKPPFSYNALIMMAIRQSPEKRLTLNGIYEFI

MKNFPYYRENKQGWQNSIRHNLSLNKCFVKVPRHYDDPGKGNYWMLDPSSDDVFIGGTTGKLRRRSTAAA

RSRLAFRRGFGVRYPAGVMEWPATDKTNCYWTTHPPANGGYSLPQHSPGFHYSPPPSSTPGFGFTSPHSS

TPQHNFSVERLLSTDTSRAAPVCSLSSTVLGTFPPLTVQTASGLLHATLPHPAGLLFPSHGHVLHDPYAS

LRTLAMSPPTAFTGTVGQSPLPLGLHLQGNVGEHGGHLTVGGTPLVLPRLTG

>CiIn_Foxg (XP_002124993.3)

MTNDAAESGHSKRENFIEMSPEYSTLIAAEDRSSVPRREDAQVPRFENIQNGGADIENGEVSPIQKDIAN

QELNDVAINMTSPQKQTNNENLEEKCPKDQKPSTSPPSNKYGKKPPYSYNALIMMAIKKSPRKRLTLSQI

YQYITTTFPYYKENKQAWQNSIRHNLSLNKCFVKVPRHYDDPGKGNYWMLDPSSDDVYIGSSTGKLRRRS

SSSQARGRLALRRRTFAQVFGSPQDILQHDPPQQIIRTDVTSRAAMLRHHDAARTMLGSGIPQRADPYPM

FQHTRFPMTTETDSRYRQLYRARLEQYYAHLASSALFGHMQATALNAQPRIVKSNPASPEPVHSHYEQTT

SPNTAPPRSDTSTPPRGIERPWASPPRRRCDVTKHETIDRDVVSRCSSESSNESSRNKREVTENSTNSPI

QQLSTRGGLPFYLTPTPNPCPNFLMPNTAGLVPNPSYPFFFPQPFHPALAFLSRPQTVASSSQL

>SaKo_Foxg1 (XP_002735197.2)

MVKIEMMAKDGGTTTTTTTTKTIGIKSSFTIDSMLPQKEKEIEDMVVNENGNLHDSDEMAEKVHDNGETM

ESNDENANHSGNRESESKKDDGNKQTGSSNGSSPPRNKYGEKPPFSYNALIMMAIRQSPEKRLTLNGIYE

FIMKHFPYYRENKQGWQNSIRHNLSLNKCFVKVPRHYDDPGKGNYWMLDPSSDDVFIGGTTGKLRRRSTS

ASRNRLAQLKRHPRLHGGGYPLQSDIKPYPMYWPASHMLPSLPQHAAASNALRYTATAGGLHTSHYNSLF

TPSPTMSRPLGSHNFSVDRLIGTDASYASPHHQNTLSPSAQTLASLRSGLGVATPTTFGLSNVTTSAVQS

GYFYPHFHHAQAATAATTYDMYSSLRGMNVSPPTAFTSTIAAQPMTTASSLLTDNTFITNNSAIGTVQVL

PRPS

>LeCa_Foxg1a

MLDMGDQRAERKAGPKTTSFTIDSLLPETVQHSLPPPPLLHHQNHNHHHHHHHHQHHHQQQQQQQQQQHHHQHHRPLSSSGRLSAVDIAAQQQQQQQQQQQQPSFRVISAVREPIVNVDGVSSGNSSGSSSSSSSSRPGSPEAGRRVDSKPATGGGAAAHGEEARPRSPERKGSVEKDIGVAVPTAAATAAGAVGSGGGETGKDGEKKNGKYEKPPFSYNALIMMAIRQSPEKRLTLNGIYEFIMTNFPYYRENKQGWQNSIRHNLSLNKCFVKVPRHYDDPGKGNYWMLDPSSDDVFIGGTTGKLRRRSTTSRGKLAFKRGARFAPAAFAFMERGSSLYWPVSPFLSLHHHHHHAQQQQQQHHHAHQPQHHHHHHHAHHQQHHHAQQHHHHIVAGGGGGGGGGGVGGGGGVVGGAAAAASALSYACAASPYTAGHHHHHHSAAAAAAAAAYGSVLSQSSLGGGGGAGGGGGVGGGSSGTAGGSGAGSLTVERLVGGDLQPYASHHLSAAAALAASVPCATAAAAAAAAAAAAGGPYGALGPCSVGLLAAGQGGYFFPHMGAAAAAAAAAAAAAAAAGGGGGGGGGGGGGSSSQGIIQGAGSPPLSQSSPHSGGLIPGCEVLRGPLSSFHAGLPGGLAGYFAQHQTGHSANSLLH

>LeCa_Foxg1b

MGDQRHAGAVKVAAKTSFSIDSLLLEAVQRGYHRGGGGDSSSSGGIIINSSSSSGIIINSSSGVVRSGDGEATGNAGGSTGVEEVEEVVEERTRSTARRDSSSGGGEKRASADANGVEGCDEADDEKSSCPRHEKSSSSSNNSKHEKPPFSYNALIMMAIRQSPEKRLTLNGIYEFIMTNFPYYRENKQGWQNSIRHNLSLNKCFVKVPRHYDDPGKGNYWMLDPCSDDVFIGGTTGKLRRRSGASRGKLAFRRGPLFASAGLALMERAASIFWPIMPPPPPPPPPPHHTHHHHHPHPSSIHPHLQNHHQQQQLQQQHGLHYSAAQTFHCPPHASAAAGLYAPLLCHAALGGPPLALTGACGGFLQLADGLRGFRDLPFHHHHHHQQQQQQLTATAFAALTGLHGGKATTEPQQQSQVDASSSTVEPCVGTSIVAAIPPALSAFYPRVSSAYASLH

>LeCa_Foxg1c

MPDMADRVTDTRALPSSKASSSFFSIARLLPDTEASRPASRASPDPDSLSCPNEPESPVITTTTATIVNNNNNNRGSVNNHAANTTVSSSSGSSGSEEEEKTDDAELAGCADDREKGAGSKASDGGGDAKEDADGSKGDKPPFSYNALIMMAIRQSPEKRLTLSGIYEYIMHNFPYYRQNKQGWQNSIRHNLSLNKCFVKVPRHYDDPGKGNYWMLDPSSDDVFIGGRTGKLRRRSAVSPRGKLQLACRRNPYLATAGLALRAPPRFSAHDLALMDRAGSLYWSVSPLLPLHSHFAGSMLGYGGASGGGGGAGGGGCPGYPASNHAAGPYSPLQLVQPQSCADAAGQPFNLSAANAQGLCLGRLGGGHQELVEHLSLQSFMQQQQHHGSAAAAAAAAAAAVAASGSTPVVPCGGSPSGCSLHSSTGANGPAASPGFIHVGPGTGYRLAGHLGLGSSALQQGLAAHHHHHHHHSTVAGHRQAHSPSLMGFPGAGLQSAYFGH

>PeMa_Foxg1a (XP_032820201.1)

MLDMGDQRAERKAGPKTSFTIDSLLPETVQHSLPPPPLLHHQNHNNHHHHHHHHHQQQQQQQQHHHQHHR

PLGSSGRLSAVDIAAQQQQPPSFRVISAAREPIVNVDDVSNSGNSSGSSSPGSPEGMRRVDSKPAAGGGA

AAAAAAHAEEPRPRSTERKGSVEKDIGVGVPTAAAAATAAATAAAAATAAAVGSGGGETGKDGDKKNGKY

EKPPFSYNALIMMAIRQSPEKRLTLNGIYEFIMTNFPYYRENKQGWQNSIRHNLSLNKCFVKVPRHYDDP

GKGNYWMLDPSSDDVFIGGTTGKLRRRSTTSRGKLAFKRGARFAPAAFAFMERGSSLYWPVSPFLSLHHH

HHHAQQQQQHHHHAHQPQHHHHHHHAHHQQHHHAQQHHHHIVAAAGGGGGGGGGGGGGVSSLGGGGGGAG

GGGGVGGGSSGTAGGSGAGSLTVERLVGGDLQPYASHHLSAAAALAASVPCAAAAAAAAAGGPYGALGPC

SVGLLAAGQGGYFFPHMGAAAAAAAAAAAAAAASSPHSAGLIPGCEVLRGPLSSFHAGLPGGLAGYFAQH

QTAHSANSLLH

>PeMa_Foxg1b (XP_032818886.1)

MGDQRHAGAVKVAAKTSFSIDSLLLEAVQRGYHRGGGGSDSSSSSIIINSSGSSGGVVRSGDGEPAGNAG

GTTGVEEVVEEVEEEEERARGTARRDSCGGEKRGNADANGAEGCDEVDDERSSCPRHEKSNNNSNNNSSN

SKHEKPPFSYNALIMMAIRQSPEKRLTLNGIYEFIMTNFPYYRENKQGWQNSIRHNLSLNKCFVKVPRHY

DDPGKGNYWMLDPCSDDVFIGGTTGKLRRRSGASRGKLAFRRGPLFASAGLALMERAASIFWPIMPPPPP

PPPPPHHPHHHHHPHPSIHPHLHNHHQQQLQQQHGLHYSAAQTFHCPPHASAAAGLYAPLLCHATLGGPP

LALAGSCGGFLQLADGLRGFRDLPFHHHHHQQQQLTATAFAALTGRHGGKATTESQQHSQVDGSSSTVEP

CVGASIVAAIPPALSAFYPRVASAYASLH

>PeMa_Foxg1c (XP_032811741.1)

MPDMADRVTDTRALPSSRASSFSIARLLPDAEASRPASRGSPDPDSLSCPTEPESPVITTTTATIFNNNN

NNRGSVNNHAANTTVSSSSSSGGGSSEEEEKTDDAEPAGCADDKEKGAGSKASDGGGESKEDGDGAKGGD

KPPFSYNALIMMAIRQSPEKRLTLSGIYEYIMHNFPYYRQNKQGWQNSIRHNLSLNKCFVKVPRHYDDPG

KGNYWMLDPSSDDVFIGGRTGKLRRRSAASPRGKLQLACRRNPYLATAGLALRAPPRFSAHDLALMDRAG

SLYWSVSPLLPLHSHFAGSMLGYGGASGGGGGGAGGGGGGCPGYPASNHAAGPYSPLQLVQPQSCADAAG

QPFNLSAASAQGLCLGRLGGGHQELVEHLGLQSFMQQQQHHHGSAAAAAAAAAAAAVAACGSTPVVPCGG

TPGSCSLHSTGANGTAASPGFIHVGPGTGYRLAGHLGLGSSALQQGLAAHHQHHHSTVAGHRQSHSPSLM

GFPGAGLQSAYFGH

>EpBu_Foxg1b (Eptbu0021100.t1)

MKEIVRGSSFTSSVKAAVSGSSFSIARLLPEAVSADASFKNDLHNEQTAITGEDASSSDPLTKSMEKLSQDEDEEEKNLEKPPFSYNALIMMAIRQSPERRLTLSGIYEHIMRRFPYYRQNCQGWQNSIRHNLSLNKCFVKVPRHYDDPGKGNYWMLDPSSDDVFIGGRSGKLRRRLTSSGRSKVLHPPCRRHHYSFETGTPTSLRIAPHFGAFAMPFLDLCGPFPWGGTSLLQPLASIITYSYRPALTPQRCPLIPRESIRIGNIQPTFGPAQHSFAPESSTRPELTPPSLCIPVRTSNSVPFPLTPSEPMYYTPSGHGVSIPRDNPHGDFRRLQVSHGGLTFGPSGVP

>EpBu_Foxg1a (Eptbu0024442.t1)

MLDMGDQREKKMIPKSSFSIDSLLPETVAPKPLVGRTSLEHPDACSLQGAERECAERDLKLDGKGGGKGGDTDERTDEKRSDEKDGEGIKEGEKKNGKYEKPPFSYNALIMMAIRQSPEKRLTLNGIYEFIMQNFPYYRENKQGWQNSIRHNLSLNKCFVKVPRHYDDPGKGNYWMLDPSSDDVFIGGTTGKLRRRSTTSRGKLAFKRGARFAPASFAFMDRTSSLYWPVSPFLSLHHANGGSLGYGCASSAYSAGAHPAYGSMLAQGSLGATSSLSGAGGSLAVERLVGSDLPYASHHLSAAAALAASVPCTSSSPYSLAPCSVGLLAGQTGYFFPHMSPSTPPTLSAAVAAATPGNGVLAGSPPPQHQPMQGHLGHLGCDALRAPLPTFPPGLPSSLASYFTQHQNATSVVPNGLLH

>ScCa_Foxg1 (XP_038642146.1)

MLDMGDKKEVKMIPKSSFSINSLVPEAVQSDNRHRSAPEQEDKNILPTLESKSENTLSKSDNSSAEPPCS

EDKEKQEEKKVDGKEGDAGKEGEKKNGKYEKPPFSYNALIMMAIRQSPEKRLTLNGIYEFIMKNFPYYRE

NKQGWQNSIRHNLSLNKCFVKVPRHYDDPGKGNYWMLDPSSDDVFIGGTTGKLRRRSTTSRAKLAFKRGA

RLTSTGLTFMDRAGSLYWPVSPFLSLHHPRASSALSYNGTTSPYPSHPMSYSSVLTQNSLANNHSFPTSN

GLSVDRLVNGEIPYATHHLTAAAALAASVPCGLSVPCSGTYSLNPCSVNLLAGQTSYFFPHVPHPSMTSQ

SSTSMTARTASSTSPQAPSSLPCESLRPSLPSFSTGLSAGLSDYFTHQNQGPSSNPLIH

>ScCa_Foxg1c (XP_038642036.1)

MGGGACLAYLGRFLNGAAEEFAVCYVCVCWWVMADLKKATVTPKRSFSIQSLLPEEVLSGEGAREPVRQE

KGKGAATEEQETELASQERSRAPGGEAEEAKGLGNSEKPPFSYNALIMMAIRQSPQKRLTLSGIYEFIVQ

NFPYYKENKQGWQNSIRHNLSLNKCFVKVPRHYDDPGKGNYWMLDPSSDDVFIGGATGKLRRRSTPVRAK

LAYKRGARLASTGLALASSLYWPMSPILSLQQPHLQGAHPLSYSPASPYPPPPYTVSLLCAQPGAHVPEL

PGYGGSQHLSPAASLPCGLSPSSCTSSPPCSPCPFNLLAGQTTYLFSQLSPLASPGAAPLSGIAQPLLKS

REFRRPPPATFPGLYFPTQNLETPFIL

>ScCa_Foxg1_like (XP_038670539.1)

MLSMGDQKQQKMVPKSTSFSIKSLLLPEAVQQSDNNQSRAGLQTVRNGDPENGAIKSDFKYQDNTESQVS

AEEDDAKKDDKKHGKYDKPPFSYNALIMMAIRQSPEKRLTLNGIYEFIMKNFPYYRENKQGWQNSIRHNL

SLNKCFVKVPRHYDDPGKGNYWMLDPSSDDVFIGGTTGKLRRRSATSRGKLAFKRGLRLSPSGLGLMDRG

NPLYWPLSPFLSLHHPHNSLNYSSAASSFLNQAPTYSSLLSGCGAGVDQMASGDLSQPLLRGSVQCGLTN

GYTVNPCSVSLWSGQAGYFFPSLHHSQSVQHQSGAGIGSPGGPSSSPPAASSLITETLRPSLTTFPSGLA

AGFTSYLSQQNGGASGNSFLH

>ScTo_Foxg1 (BBG57206.1)

MLDMGDKKEVKMIPKSSFSINSLVPEAVQSDNRHRSAPEQEDKNILPTLESKSENTLSKSDNSSAEPPCS

EDKEKQEEKKVDGKEGDAGKEGEKKNGKYEKPPFSYNALIMMAIRQSPEKRLTLNGIYEFIMKNFPYYRE

NKQGWQNSIRHNLSLNKCFVKVPRHYDDPGKGNYWMLDPSSDDVFIGGTTGKLRRRSTTSRAKLAFKRGA

RLTSTGLTFMDRAGSLYWPVSPFLSLHHPRASSALSYNGTTSPYPSHPMSYSSVLTQNSLANNHSFPTSN

GLSVDRLVNGEIPYATHHLTAAAALAASVPCGLSVPCSGTYSLNPCSVNLLAGQTSYFFPHVPHPSMTSQ

SSTSMTARTASSTSPQAPSTLPCESLRPSLPSFSTGLSAGLSDYFTHQNQGPSSNPLIH

>ScTo_Foxg2 (BBG57207.1)

MADLKKATVTPKRSFSIQSLLPEEVLSGEGAREPLRQEKGKSAAAAEQEAELASQERRRAPGGDAEEEKG

LGNPEKPPFSYNALIMMAIRQSPQKRLTLSGIYEFIVQNFPYYKENKQGWQNSIRHNLSLNKCFVKVPRH

YDDPGKGNYWMLDPSSDDVFIGGATGKLRRRSTPVRAKLAYKRGARLASTGLALASSLYWPLSPLLSLQQ

PHLQGAHPLSYSPASPYPAPPYTVSLLCAQPGAHMPEVPGYGGSQHLSPTTLAASLPRGLSPSSSSCTSS

PPCSPCALNLLAGQTTYLFSQLSPLAGPGGAPLSGIAPPLLKSREFRRPPPATFPGLYFPTQNLETPFIL

>ScTo_Foxg3 (scf_scyto00013220)

MLSMGDQKQPKMVPKSTSFSIKSLLLPEAVQQSDNNQSRAGLQTVRNGDPENGAIKSDFKYQDTAESQVSAEEDDPKKDDKKHGKYDKPPFSYNALIMMAIRQSPEKRLTLNGIYEFIMKNFPYYRENKQGWQNSIRHNLSLNKCFVKVPRHYDDPGKGNYWMLDPSSDDVFIGGTTGKLRRRSATSRGKLAFKRGLRLSPSGLGLMDRGNPLYWPLSPFLSLHHPHNSLNYSSAASSFLNQAPTYSSLLSGCGSGVDQMASGDLSHPLLRGSVQCGLANGYTVNPCSVSLLSGQAGYFFPSLHHSQSVQHQSGAGMGNPGAPSSSPPAASSLITDTLRPSLSTFPSGLAAGFTSYLSQQNGGASGNSFLH-

>RhTy_Foxg1 (XP_020380787.1)

MLDMGDKKEVKMIPKSSFSINSLVPEAVQSDSRQRAGPEHDDKPCLPALDTKPENAGGKSESSSADPVGS

DDKDKPEEKKADGKEADAGKEGEKKNGKYEKPPFSYNALIMMAIRQSPEKRLTLNGIYEFIMKNFPYYRE

NKQGWQNSIRHNLSLNKCFVKVPRHYDDPGKGNYWMLDPSSDDVFIGGTTGKLRRRSTTSRAKLAFKRGA

RLTSTGLTFMDRAGSLYWPVSPFLSLHHPRASSTLSYNGTTSPYPSHPMSYSSVLTQNSLANNHSFPTSN

GLSVDRLVNGEIPYATHHLTAAAALAASVPCGLSVPCSGTYSLNPCSVNLLAGQTSYFFPHVPHPSMTSQ

SSTSMTARTASSTSPQAPSTLPCESLRPSLPSFSTGLSGGLSDYFTHQNQGPSSNPLIH

>RhTy_Foxg1-like (XP_020371701.1)

MLNMGDQKQPKMVPKCTSFSIKSLLLPEAAQQQQQQSDNNQGSRVGVPELKADFKYQDSPETQLGPAEEE

EEDEHKKAEKKHGKYDKPPFSYNALIMMAIRQSPDKRLTLNGIYEFIMKNFPYYRDNKQGWQNSIRHNLS

LNKCFVKVPRHYDDPGKGNYWMLDPSSDDVFIGGTTGKLRRRSATSRGKLAFKRGLRLSPSGLGLMDRGN

PLYWPLSPFLSLHHPHNSLNYSSAASSFLNQAPTYSSLLSGCGAGIDQMASGDLSHPLLRGSVQCGLTNG

YSVNPCSVSLLSGQTGYFFPSLPHSQSVQHQNGTGMGNPGAPSSSPPAASPLITETLRPSLSTFPSGLAA

GFTSYLSQQNGGTASNSFLH

>RhTy_Foxg1_ (CM038836.1)

MLDMAGLEKAETTKKFSFSIESSLPEERPEQNEGAGGSGKDQEPEERQEKPPFSYNALIMMAIRQSPGKRLTLSGIYEFIIRNFPYYKDNKQGWQNSIRHNLSLNKCFVKVPRHYDDPGKGNYWMLDPSSDDVFIGGSTGKLRRRSAPSRARLACKRGTRTGLTLASSFYWPTHPFSPWQQTPQPYLGLPPPPPPPPPYLSVLSQSSTHHHGGTQVLYGAPHVQLTAAMATTLPCPMPCALNLVTGNTSYLFSQFSPVAPLMGGLGPPTLKGNELQRPSATYLSTQNLASPFIL-

>CaCa_Foxg1 (XP_041071115.1)

MLDMGDKKEVKMIPKSSFSINSLVPEAVQSDNRHRSAPEEQDKNILPAVESKSENTLSKSENSSAEPPCS

EEKEKQEDKKADGKEGDAGKEGEKKNGKYEKPPFSYNALIMMAIRQSPEKRLTLNGIYEFIMKNFPYYRE

NKQGWQNSIRHNLSLNKCFVKVPRHYDDPGKGNYWMLDPSSDDVFIGGTTGKLRRRSTTSRAKLAFKRGA

RLTSTGLTFMDRAGSLYWPVSPFLSLHHPRASSTLSYNGTTSPYPSHPMSYSSVLTQNSLANNHSFPTSN

GLSVDRLVNGEIPYATHHLTAAAALAASVPCGLSVPCSGTYSLNPCSVNLLAGQTSYFFPHVPHPSMTSQ

SSTSMTARTASSTSPQAPSTLPCESLRPSLPSFSTGLSGGLSDYFTHQNQGPSSNPLIH

>CaCa_Foxg1-like (XP_041038727.1)

MLSMGDQKQPKMVPKSTSFSIKSLLLPEAVQQSDNQNRTGLQSNKNGDPENGTIKSDFKYQDNPETQVSA

EEDDPKKDDKKHGKYDKPPFSYNALIMMAIRQSPEKRLTLNGIYEFIMKNFPYYRENKQGWQNSIRHNLS

LNKCFVKVPRHYDDPGKGNYWMLDPSSDDVFIGGTTGKLRRRSATSRGKLAFKRGLRLSPSGLGLMDRGN

PLYWPLSPFLSLHHPHNSLNYSSAASSFLNQAPTYSSLLSGCGSGVDQMASGDLSHPLLRGSVQCGLTNG

YTVNPCSVSLLSGQAGYFFPSLHHSQSVQHQNGTGMGNPGAPSSSPPAASSLITETLRPSLSTFPSGLAA

GFTSYLSQQNGGASSNSFLH

>CaCa_Foxg1c (XP_041035147.1)

MLDMASLKDAKTVPKRNFSIQSLLPEAISGDGPGGGAGAAAAAVAGSAAGAEEGTPSGSLKGGGAAEREP

DFPGKEERRGQAAGDGEEAKGMGKFQKPPFSYNALIMMAIRQSPGKRLTLSGIYEFIVQNFPYYRENKQG

WQNSIRHNLSLNKCFVKVPRHYNDPGKGNYWMLDPSSDDVFIGGTTGKLRRRATPARVKLAFKRGTRLAS

AGLALAGSLCWPVSPLLPLQQPPTQSGYPLGYNCPPPYLEHPAPYAPLLPHGAHQRGAASLSCGLSLPAS

CSSPCPLNLLAGQTAYLLSQLSPVTGPSGPLGGMGPALLKGSSEFRRPAASFPGAYFPNRNLEPPFIL

>StFa_Foxg1 (XP_048392980.1)

MLDMGDKKEVKMIPKSSFSINSLVPEAVQSDSRQRAGPEHDDKPCLPALDTKPENAGGKSESSSADPVGS

DDKDKPEEKKADGKEADAGKEGEKKNGKYEKPPFSYNALIMMAIRQSPEKRLTLNGIYEFIMKNFPYYRE

NKQGWQNSIRHNLSLNKCFVKVPRHYDDPGKGNYWMLDPSSDDVFIGGTTGKLRRRSTTSRAKLAFKRGA

RLTSTGLTFMDRAGSLYWPVSPFLSLHHPRASSTLSYNGTTSPYPSHPMSYSSVLTQNSLANNHSFPTSN

GLSVDRLVNGEIPYATHHLTAAAALAASVPCGLSVPCSGTYSLNPCSVNLLAGQTSYFFPHVPHPSMTSQ

SSTSMTARTASSTSPQAPSTLPCESLRPSLPSFSTGLSGGLSDYFTHQNQGPSSNPLIH

>StFa_Foxg1-like (XP_048392049.1)

MLNMGDQKQPKMVPKCTSFSIKSLLLPEAAQQQQQQQRDGNQGSRAGDPDLKADFKYQDSPETQLGPAEE

EEEDEHKKGEKKHGKYDKPPFSYNALIMMAIRQSPDKRLTLNGIYEFIMKNFPYYRDNKQGWQNSIRHNL

SLNKCFVKVPRHYDDPGKGNYWMLDPSSDDVFIGGTTGKLRRRSATSRGKLAFKRGLRLSPSGLGLMDRG

NPLYWPLSPFLSLHHPHNSLNYSSAASSFLNQAPTYSSLLSGCGAGVDQMASGDLSHPLLRGSVQCGLTN

GYTVNPCSVSLLSGQTGYFFPSLHHSQSVQHQNGTGMGNPGAPSSSPPAASPLITETLRPSLSTFPSGLA

AGFTSYLSQQNGGAASNSFLH

>StFa_Foxg1-like’ (XP_048378260.1)

MLDMAGLEEAEITKKSSLSIESLLPEERPEQSEGAGGSGKDQEPEERQEKPPFSYNALIMMAIRQSPGKR

LTLSGIYEFIIRNFPYYKDNKQGWQNSIRHNLSLNKCFVKVPRHYDDPGKGNYWMLDPSSDDVFIGGSTG

KLRRRSAPSRARLAYKRGTRAGLTLASSFYWPTPPFSPWQHNPLPYLELPPPPPLPPPYLSVLSQSSTHH

PEGAQVVYGAPHVQLTAAATTTLPCPMPCALNLVTSNASYLFSQFSPVTPLMGGLGPPTLKGKELQRPSA

TFPGAYLSTQNLGSPFIL

>PrPe_Foxg1 (XP_051880924.1)

MLDMGDKKEVKMIPKSSFSINSLVPEAVQSDTRHRAAPEDEDKNILPAVESKSENLLGKSDGTSAEPPCA

EDKDKQEEKKAEGKEGDGAKEGEKKNGKYEKPPFSYNALIMMAIRQSPEKRLTLNGIYEFIMKNFPYYRE

NKQGWQNSIRHNLSLNKCFVKVPRHYDDPGKGNYWMLDPSSDDVFIGGTTGKLRRRSTTSRAKLAFKRGA

RLTSTGLTFMDRAGSLYWPVSPFLSLHHPRASSTLSYNGTTSPYPSHPMSYSSVLTQNSLANNHSFPTSN

GLSVDRLVNGEIPYATHHLTAAAALAASVPCGLSVPCSGTYSLNPCSVNLLAGQTSYFFPHVPHPSMTSQ

SSTSMTARTASSTSPQAPSTLPCESLRPSLPSFSTGLSGGLSDYFTHQNQGPSSNPLIH

> PrPe_Foxg1-like (XP_051867297.1)

MGDEKQPKMVPKSTSFSIKNLLLPEEVQQSDNSQNARAGFLSSKSGDIESGAVKSDFKYQDHPETQVVTE

GSEQKKDEKKQGKYDKPPFSYNALIMMAIRQSPEKRLTLNGIYEFIMKNFPYYRENKQGWQNSIRHNLSL

NKCFVKVPRHYDDPGKGNYWMLDPSSDDVFIGGTTGKLRRRSATSRGKLAFKRGLRLSTSGLGLVERGNP

LYWPLSPFLSLHHPHNGLNYSSAASSFLNQAPTYSSLLSGCGSGVDQMASGDLSHPLLRGSVQCGLANGY

TVNPCSVSLLSGQAGYFFPSLHHTQTVQHQNGPGMGNPGAPSSSPPAASLLSETLRPSLSTFPSGLAAGF

TSYLSQQNGGAPGNSFLH

> PrPe_Foxg1-like’ (XP_051900007.1)

MLAMGDLKEPRVIHRASFAIKNLLPEAMVSESPELEVGRDPGGPSGVGTARARDVPGKEADQEGGRDGEG

NTESVRHEKPPFSYNALIMMAIRQSPDKRLTLSAIYEFIMQNFPYYKENKQGWQNSIRHNLSLNKCFVKV

PRHYDDPGKGNYWMLDPSSDDVFIGGATGKLRRRSTSTRAKLAFKRGARLASPGLALAGSLYWPMSPLLS

LHQAHPLSLNAAASSSYPDHAASYAAVLSQQLGAGGDKLAALDLPFGASHHQVTAAALATSLPHRLAIPA

SYRPCSVNLLAGQASYFISQFPHPPVTGPAASLGGIAPPLKHSEEFGRPYPSFPNTFFPSQTQNSPFNPL

FLKSTTICLKVG

>AmRa_FoxG1 (XP_032882871.1)

MLDMGERKEVKMIPKSSFSINSLVPEAVQSDIRHRAAPEDQDKNILPAVDSKSENLLSKSDSSAEPPCSE

DKDKQEEKKAEGKEGEPGTKEGEKKNGKYEKPPFSYNALIMMAIRQSPEKRLTLNGIYEFIMKNFPYYRE

NKQGWQNSIRHNLSLNKCFVKVPRHYDDPGKGNYWMLDPSSDDVFIGGTTGKLRRRSTTSRAKLAFKRGA

RLTSTGLTFMDRAGSLYWPVSPFLSLHHPRASSALSYNGTTSPYPSHPMSYSSMLTQNSLASNHAFPTSN

GLSVDRLVNGEIPYATHHLTAAAALAASVPCGLSVPCSGTYSLNPCSVNLLSGQTSYFFPHVPHPSMTSQ

SSTSMTARTASSTSPQASSTLPCEALRPSLPSFTTGLSGGLSDYFTHQNQGPSSNPLIH

>AmRa_Foxg1-like (XP_032881316.1)

MGDEEQPKMVPKSSSFSIKNLLLPEDFHQNESNHRSDFPSGKGGETESGAVKADFKYQERPETQVVSEGG

DQKKEEKKQGKYDKPPFSYNALIMMAIRQSAEKRLTLNGIYEFIMRNFPYYRENKQGWQNSIRHNLSLNK

CFVKVPRHYDDPGKGNYWMLDPSSDDVFIGGTTGKLRRRSVTSRGKLAFKRGLRLSTTGLGLVERSNPLY

WPLSPFLSLHHPHNTLNYSSAASSFLNQAPSYSSLLTGCGSGVEQMASGDISHPLLRGSVQCGYTVNPCS

VSLLAGQAGYFFPSLHHTQAVQQQNGPGMGNPGAPSSSPPLATSILSETLRPSLSTFPSGLAAGFTSYLS

QQNAGAPNNAFLH

> AmRa_Foxg1-like’ (XP_032870162.1)

MEDRNEAGVVHRRPFTIKNLLPDSVAGEELEVKAEGDKEGGQDAAGALQSRDVPGKEGGDGEKEEGEKYE

KPPFSYNALIMMAIRQSRDKRLTLSSIYEFIVGNFPYYKDNKQGWQNSIRHNLSLNKCFVKVPRHFDDPG

KGNYWMLDPSSDDVFIGGSTGKLRRRATTTRAKLAFRRGCRLTSPGLALAGSLYWPVSPLLSLHQAHPGP

AFSFAASPPFPDQCPPYAAVLSHQLGPGAERLAALGDLPYGPAHRQVSAAATALAACSPATYRACPVNLL

SGQASYYFTPLPLPAASLTGVNPPLLKHSEDFGTRNAGFLCPYFPNPTPNPFSPLVL

>CaMi_Foxg1 (XP_007891603.1)

MLDMGDKKEVKMIPKSSFSINSLVPEAVQSDNHHHHRSAPDDEDKNLLPAVESKSENVPSKTDSSSGEQP

SAEEKEKQEEKKADGKEGDGGKEGEKKNGKYEKPPFSYNALIMMAIRQSPEKRLTLNGIYEFIMKNFPYY

RENKQGWQNSIRHNLSLNKCFVKVPRHYDDPGKGNYWMLDPSSDDVFIGGTTGKLRRRSTTSRAKLAFKR

GARLTSTGLTFMDRAGSLYWPVSPFLSLHHPRASSALSYNGTTSAYPSHPMSYSSVLTQNSLANSHSFPT

SNGLSVDRLVNGEIPYATHHLTAAAALAASVPCGLSVPCSGTYSLNPCSVNLLAGQTSYFFPHVPHPSMT

SQSSTSMSARAASSTSPQAPSTLPCESLRPSLPSFSTGLSGGLSDYFTHQTQGSSSNPLIH

> CaMi_Foxg1-like (XP_007896671.1)

MLSMGDQKQPKMVPKTTSFTIKSLLLPEADSQGARVGLQSVRNCAPELGSSSSSSNSSKAEQSGGGGGGG

GKAEFKSQSDPERAEGGSGSREESPEEEEEEEETRKREDKRQSKYDKPPFSYNALIMMAIRQSPEKRLTL

NGIYEFIMKNFPYYRENKQGWQNSIRHNLSLNKCFVKVPRHYDDPGKGNYWMLDPSSDDVFIGGTTGKLR

RRSATSRGKLAFKRGLRLSPSGLGLMDRGNPLYWPLSPFLSLHPPHGSLGYSSAASSFLNQAPTYSSLLS

GCGSGVDQMANGDLSHSLLRGSVQCSLTNSYSVSPCSVSLLAGQTGYFFPSLHHAQSVHQTGSGMGNPGG

ASSSSSSSPPAASALIADALRPTLSTFPSGLAAGFSSYLSQQNGATSSNSFLH

>AcRu_Foxg1-like (XP_033887769.1)

MLDMGERKEVKMIPKSSFSINSLVPEAVQSDNHHRSAPEEQEKHLLPNPVQDSKPENIDNTNENSSPELP

CSEEKEKQEEKKVECKEGDGGKDGEKKNGKYEKPPFSYNALIMMAIRQSPEKRLTLNGIYEFIMKNFPYY

RENKQGWQNSIRHNLSLNKCFVKVPRHYDDPGKGNYWMLDPSSDDVFIGGTTGKLRRRSTTSRAKLAFKR

GARLTSTGLTFMDRAGSLYWPMSPFLSLHHPRASSALSYNGTTAAYPSHPMSYSSLLTQNTLGNNHSFSA

TNGLSVDRLVNGDIPYATHHLTAAALAASVPCGLSVPCSGTYSLNPCSVNLLSGQASYFFPHVPHPSMTS

QSSTSMTARAASSTSPQAPSSLACESLRPSLPSFTTGLSGGLSDYFPHHSQGSTSNPLIH

> AcRu_Foxg1-like’ (XP_033892910.1)

MLDMGERKEVKMIPKSSFSINSLVPEAVQSDNHHRSAPEEHEKHLLPNPVQDSKPENIDNTNENSSPELP

CSEEKEKQEEKKVECKEGDGGKDGEKKNGKYEKPPFSYNALIMMAIRQSPEKRLTLNGIYEFIMKNFPYY

RENKQGWQNSIRHNLSLNKCFVKVPRHYDDPGKGNYWMLDPSSDDVFIGGTTGKLRRRSTTSRAKLAFKR

GARLTSTGLTFMDRAGSLYWPMSPFLSLHHPRASSALSYNGTTAAYPSHPMSYSSLLTQSTLGNNHSFSA

TNGLSVDRLVNGDIPYATHHLTAAALAASVPCGLSVPCSGTYSLNPCSVNLLSGQASYFFPHVPHPSMTS

QSSTSMTARAASSTSPQAPSSLACESLRPSLPSFTTGLSGGLSDYFPHHSQGSTSNPLIH

> AcRu_Foxg1b (XP_033859035.1)

MGDQKEPTMVPKSTSFSIKSLLFPSKSDNPNSGAPEKKTLPEEMESCYFNQDNHREEEADGPKEQNKNGK

YDKPAFSYNALIMMAIRQSPEKRLTLNGIYEFIMNNFPYYREHKQGWQNSIRHNLSLNKCFVKVPRHYDD

PGKGNYWMLDPSSDDVFIGGTTGKLRRRSTTSRGKLALKKGLRFGPLGLGLSDRASNPLYWQISPFLSLH

HPHYNRSASGFLSQGHAYGSLLSGIDQLGNGDLSRPILGGSGLTNSYGVSTSSVSLLSGQSGYFVSGAQH

SQPVQHNGGGFGVPSSSPSTLISETLRPSLPSFTSGVSTGFAGVLSHQNRVTSASSFLN

> AcRu_Foxg1-like’’ (XP_033873148.2)

MGDQTEPTMVPKSTSFSIKSLLFLKSDNPSLGAPEKKNLPQGSDSAKPQDPEEMESCFNQENHKEEEEGE

RPKEQNKNAKYDKPPFSYNALIMMAIRQSPEKRLTLNGIYEFIMNNFPYYREHKQGWQNSIRHNLSLNKC

FVKVPRHYDDPGKGNYWMLDPSSDDVFIGGTTGKLRRRSATSRGKLALKRGLRFTPLGLGFNDGASNPLY

WQISPFLSLHPPHYNGSASGFLRQGHAYTSLLSGVDQLANGDLSRPILGGSGLTNSYGVSTSSVSLLSGQ

SGYFVSGAQHSQPVQHNGSGFGVPSSSPPTLISETLRPSLPSFTPGVSTGFAGVLSHQNRVTSASSFLN

>AcRu_foxg1-like’’’ (XP_034767013.2)

MGLRAAGRTAPSLSLPLSFMLSSCDSVDLVGCEMLDMERLKEAPGIVRASSFSIKSLLQEVAGSDSGAPD

SGAHADTSRGLETQRTGSPHPGVAERDGGRHDNTNNSNNNNNNNNEDKEGERGGDKKTKGSEQDREGEKA

ATCDKPPFSYNALIMMAIRQSPEKRLTLNGIYQFIVQNFPYYKENKQGWQNSIRHNLSLNKCFLKVPRHY

DDPGKGNYWMLDPSSDDVFIGGTTGKLRRRSTASSRAKLAFKQGNRLASSAAAAAAAAAGLAFAGSLYWP

VPPFLSLQQPHPHHQPGSTLGYSSSYFGHPSSYAASILSQTSHQLSATAAGVDRLLQGAAESSYAGTAVG

SHHHHQVTAAASFAASSLPCGLSLPTSLNPCSFNLLAAGRASYFFSHHVPHHPAVQGTQPLPAPLESSPL

KGFPVEQHLHCWSGSSTAGDPGHFTQNHQTSSFNSLLH

>PoSp_Foxg1 (XP_041131479.1)

MLDMGERKEVKMIPKSSFSINSLVPEAVQSDNHHRSAPEEQEKHLLPNPVQDSKPENIDNTNENSSSELP

CSEEKEKQEDKKVECKEGDSGKDGEKKNGKYEKPPFSYNALIMMAIRQSPEKRLTLNGIYEFIMKNFPYY

RENKQGWQNSIRHNLSLNKCFVKVPRHYDDPGKGNYWMLDPSSDDVFIGGTTGKLRRRSTTSRAKLAFKR

GARLTSTGLTFMDRAGSLYWPMSPFLSLHHPRASSALSYNGTTAAYPSHPMSYSSLLTQNTLGNNHSFSA

TNGLSVDRLVNGDIPYATHHLTAAALAASVPCGLSVPCSGTYSLNPCSVNLLSGQASYFFPHVPHPSMTS

QSSTSMTARAASSTSPQAPSSLACESLRPSLPSFTAGLSGGLSDYFPHHSQGSTSNPLIH

>PoSp_Foxg1-like (XP_041121395.1)

MLDMGERKEVKMIPKSSFSINSLVPEAVQSDNHHRSAPEEQEKHLLPKPVQDSKPENIDNTNENSSSELP

CSEEKEKQEDKKVECKEGDGGKDGEKKNGKYEKPPFSYNALIMMAIRQSPEKRLTLNGIYEFIMKNFPYY

RENKQGWQNSIRHNLSLNKCFVKVPRHYDDPGKGNYWMLDPSSDDVFIGGTTGKLRRRSTTSRAKLAFKR

GARLTSTGLTFMDRAGSLYWPMSPFLSLHHPRASSALSYNGTTAAYPNHPISYSSLLTQNTLGNNHSFSA

TNGLSVDRLVNGDIPYATHHLTAAALAASVPCGLSVPCSGTYSLNPCSVNLLSGQASYFFPHVPHPSMTS

QSSTSMTARAASSASPQAPSSLACESLRPSLPSFTAGLSGGLSDYFPHHSQGSTSNPLIH

>PoSp_Foxg1b (XP_041106931.1)

MGDQKEPTMVPKSTSFSIKSLLFPSKSDNPNSRTPEKKTVTEEMESCSFNQDNHGEEEADGPKEQNKNEK

YDKPPFSYNALIMMAIRQSPEKRLTLNGIYEFIMNNFPYYREHKQGWQNSIRHNLSLNKCFVKVPRHYDD

PGKGNYWMLDPSSDDVFIGGTTGKLRRRSTTSRGKLALKKGLRFGPLGLGLNDRASNPFYWQISPFLSLH

HPHYNRSASGFLSQGHAYGSLLSGIDQLGNGDLTRPILGGSGLTNSYGVSASSVSLLSGQAGYFVSGAQH

SQSVQHSGGGFGVPSSSPSTLISETLRPSLPSFTSGVSTGFAGVLSHQNRVTSASSFLN

>PoSp_Foxg1-like’ (XP_041107541.1)

MGDQKEPTVVPKSTSFSIKSLLFPKSDNPNSGPPEKKTLPQGLDPANPRDPEQMESSFNQDNHREEGADG

PKELNKNAKYDKPPFSYNALIMMAIRQSPEKRLTLNGIYEFIMNNFPYYREHKQGWQNSIRHNLSLNKCF

VKVPRHYDDPGKGNYWMLDPSSDDVFIGGTTGKLRRRSATSRGKLALKRGLRFAPLGLGFNDGASNPLYW

QISPFLSLHPPHYNGSASGFLRQGHAYGSLLSGVDHLANGNLSRPILGGSGLTNSYGVSTSSVSLLSGQA

GYFVTGAQHSQSVHHNGSGFGVPSSSPPTLISETPRLHSFTPSVSTGFAGVLSHQNRVTSASSFLN

>PoSp_Foxg1c (XP_041090454.1)

MERLKEAPGTVRTSSFSIKSLLQEGAGSGCGAPDSGAHGDTSRALETQRTGSPQAGGVERDNINNSNNKI

NNEDEEEERGGDEKTKGLDQDRAGEKAAKCDKPPFSYNALIMMAIRQSPEKRLTLNGIYQFIVQNFPYYK

ENKQGWQNSIRHNLSLNKCFLKVPRHYDDPGKGNYWMLDPSSDDVFIGGTTGKLRRRSTSSSRAKLAFKQ

GNRLSSSAAAAAAGLAFAGSLYWPVPPFLSVQQPHPHHHPGSTLGYSSSYFGHPSSYATSILSQTSHQLS

ATAAGVDRLLQGAAESSYTGTAGGSHHRHQVAAAASFAASSLPCGLSLPTSMTPCSFNLLAAGQASYFFS

HHVPHHPAVQGTQPPPAPLQSSPPKGSPVEQHLHCWSGSSTAGDFPGCFTQNHQTSSINSLLH

>LeOc_Foxg1 (XP_006632231.1)

MLDMGERKEVKMIPKSSFSINSLVPEAVQSDNHHRSAPEEEEKNPLPAQVQDTKSESVCPRSENTSAESA

CTEEKDKQEEKKDCKEGDSAKDGEKKNGKYEKPPFSYNALIMMAIRQSPEKRLTLNGIYEFIMKNFPYYR

ENKQGWQNSIRHNLSLNKCFVKVPRHYDDPGKGNYWMLDPSSDDVFIGGTTGKLRRRSTTSRAKLAFKRG

ARLTSTGLTFMDRAGSLYWPMSPFLSLHHPRASSALSYNGTTSAYPSHPMSYSSVLTQNTLGNNHSFPAS

NGLSVDRLVNGEIPYATHHLTAAALAASVPCGLSVPCSGTYSLNPCSVNLLAGQTSYFFPHVPHPSMTSQ

SSTSMTARAASSTSPQAPSSLPCESLRPSLPSFTTGLSGGLSDYFTHQNQGSTSNPLIH

>LeOc_Foxg1-like (XP_006638532.1)

MGDHKEPTMVPKSTSFSIKSLLLPSKCDNPGSGAPEKRTVPPGSDPAKPPAPTEMDLESPSLENHEEDDE

GGDRPAEQSKTGKYDKPPFSYNALIMMAIRQSPEKRLTLNGIYEFIMKNFPYYREHKQGWQNSIRHNLSL

NKCFVKVPRHYDDPGKGNYWMLDPSSDDVFIGGSTGKLRRRSATSRGKLAMKRGLRFAPLGLGLNDRANN

PLYWQISPFLSLHHPHYNGSASGFLNQGHAYGSLLSGVESLGNGDLSRPILGGPAGGIGLTSSYNVNTSP

VSLLSGQTGYFLSGTQHVQPLQQSGSRFGAPSSSPPTLLTESLRTSLPSFAQGVSTGFPGVASHHNRATP

ANSFLN

>LeOc_Foxg1-like’ (XP_015196379.1)

MRIQSAIRTESARRGTTAKPRDEDEALFKKIPVQLAVLLGWTAAPRLPHKSPFSIKSLLQEEAMSDGAEG

SGGTGQPEREPPPPPPPAREEPGEARDGQDSREGRGACRAPEEAAEGEEQREARHEKPPFSYNALIMMAI

RQSPDKRLTLNGIYEFIMRNFPYYKENKQGWQNSIRHNLSLNKCFVKVPRHYDDPGKGNYWMLDPSSDDV

FIGGTTGKLRRRSSASSRAKLAFKRGGRLPPPAAGLAFAGSLYWPVPPFLSLQRPEHPGYTPPYFAPHAA

ALAQAPRPVCAADPSFLGSGGHSLHHHHHHQVAAASFAASSLPCGLSPCSLNLLAGQASYFFSRHVPRPP

TPPPGASPQKSPALGQGLHARGGSSYVAGLSAVEFPNYFTQGRPGSPFNSALH

>AmCa_Foxg1 (MBN3302885.1)

MLDMGERKEVKMIPKSSFSINSLVPEAVQSDNQHRAEDEESHPVPEPGQDPKSESCKSEHSSPESGCTED

KDKPEEKKDCKEGDAAKDGEKKNGKYEKPPFSYNALIMMAIRQSPEKRLTLNGIYEFIMKNFPYYRENKQ

GWQNSIRHNLSLNKCFVKVPRHYDDPGKGNYWMLDPSSDDVFIGGTTGKLRRRSTTSRAKLAFKRGARLT

STGLTFMDRAGSLYWPMSPFLSLHHPRASSALGYNGTASAYPSHPIVDRLVNGDIPYATHHLTAAALAAS

VPCGLSVPCSGTYSLNPCSVNLLAGQTSYFFPHVPHPSMTSQSSTSMTARAASSSSPQAPSSLPCESLRP

SLPSFTTGLSGGLSDYFTHQNQGSTSNPLIH

>AmCa_Foxg1’ (MBN3309059.1)

MGDQREPTMVPKSTSFSIKSLLLPAKCDNPGSGAPEKRTLPPGSESGTSLDPTDMETSSLTQENHQEDEE

ASDRAKKPSKHGKYEKPPFSYNALIMMAIRQSPEKRLTLNGIYEFIMKNFPYYREHKQGWQNSIRHNLSL

NKCFVKVPRHYDDPGKGNYWMLDPSSDDVFIGGTTGKLRRRSATSRGKLAMKRGLRFAPLGLGLNERASN

PLYWQISPFLSLHHPHYNGSTAGFLNQGHAYGSILSGVEQLGNGDLTRPILGGSAGGIGLTNGYGVNTSP

VGLLSGQAGYFVSGTQHTQALSQSGAGFGVPSSSSPTLLSETLRTSLPSFTPGVSTGFSGVISHPNRVAP

ANTFLN

>AmCa_Foxg1’’ (MBN3305475.1)

MMAIRQSPDKRLTLNGIYEFIIHNFPYYKENKQGWQNSIRHNLSLNKCFVKVPRHYDDPGKGNYWMLDPS

SDDVFIGGTTGKLRRRSTASSRAKLAFKRGSRLASSAAGLAFAGSLYWPVPPFLSLQQPSPHPGYSSSYF

GPHGTYAASVLSQTSHQLSAAAAGVDRLLQGGAESSYGGPSGHHHHHQVTAAASFAASSLPCGLSLPSTL

NPCSFNLLAGQASYFFSHHVPHPAAHGPQPPAPPSPASPPKVSPVGQLLHGRSGSSYVGSLSASEFPNYF

TQSNQGSPFNALLH

>PoSe_ Foxg1 (XP_039598003.1)

MLDMGERKEVKMIPKSSFSINSLVPEAAQSDNHHRSVPEAEEKNLLQNPGQDAKSESAGNKNEGGSTELS

CSEDKEKADEKKVDCKEGDSGKDGEKKNGKYEKPPFSYNALIMMAIRQSPEKRLTLNGIYEFIMKNFPYY

RENKQGWQNSIRHNLSLNKCFVKVPRHYDDPGKGNYWMLDPSSDDVFIGGTTGKLRRRSTTSRAKLAFKR

GARLTSTGLTFMDRAGSLYWPMSPFLSLHHPRASGALSYNGTTSAYPSHPMSYSSVLTQNTLGNNHSFSA

SNGLSVDRLVNGEIPYATHHLTAAALAASVPCGLSVPCSGTYSINPCSVNLLAGQTSYFFPHVPHPSMTS

QSSTSMTARAASSASPQAPSTLPCESLRPSLPSFTTGLSGGLSDYFTHQNQGSTSNPLIH

>PoSe_ Foxg1b (XP_039594638.1)

MGDQKEPTMVPKSTSFSIKSLLLPSKSDKASSGSLAKGRLPAGSETAKPAATKEMASSPEKQECRLEEEE

SADQPKEPNKNGKYDKPPFSYNALIMMAIRQSPEKRLTLNGIYEFIMKNFPYYREHKQGWQNSIRHNLSL

NKCFVKVPRHYDDPGKGNYWMLDPSSDDVFIGGTTGKLRRRSATSRGKLALKRGLRFAPLGLGLNERSTN

PLYWQISPFLSLHHAHYNGSASGFLNQGHAYGSFLSGMDQLSNGELPRPILASTAGGISLTNTYGVSTSS

VSLLSGQAGYFVSGAQHSQSVQPSGGTGGFGVPSTSPSTLISETVGPALPPFTQGVSAAFSGVLSHQNRV

TSADSFLN

>PoSe_ Foxg1c (XP_039626036.1)

MPDMETLKETPGSVYKSPFSINSLLRERPLNDGAVNQTRDKASPFHLESSDSPRDPIHGESPLKEWGICA

NGLTSSAKELVENLIKGAECSSDWVADTKELGDEGAKGQANGSKDFTRGASLNCENVDVTEPSEKGVLSE

GATKGEKETKHDKPPFSYNALIMMAIRQSPEKRLTLNGIYEFIIQNFPYYKENKQGWQNSIRHNLSLNKC

FVKVPRHYDDPGKGNYWMLDPSSDDVFIGGTTGKLRRRSTASSRAKMGFKRGNRLASSAAAAAAASLAFA

GSLYWPMSPFISLQQTPHPRHAGTPLGYTFGSPSSSYAAASVLSQASQQLSSSGVDRLIQGSGEPSYTLP

HGHHHQVTAAASFSAASLPCTIPLSTSLNPCSFNLLATGQTSYFFSHHIPHPNGSGTIPTSQMPHATTSP

PKLSPVEQFLHASNGSNFSGMSAASDFSGYFTQNHGSSFNGILH

>DaRe_Foxg1 (NP_571142.1)

MLDMGERKEVKMIPKSSFSINSLVPEAVQSDNHHHHHHQQQQHHHRTVHEEEEKTPLPAQVQEQKSENTC

AKSDNSSHDSSSTDEKEKQEEKRDAKEGEGGKEGDKKNGKYEKPPFSYNALIMMAIRQSPEKRLTLNGIY

EFIMKNFPYYRENKQGWQNSIRHNLSLNKCFVKVPRHYDDPGKGNYWMLDPSSDDVFIGGTTGKLRRRST

TSRAKLAFKRGARLTSTGLTFMDRAGSLYWPMSPFLSLHHPRASSALSYNGASSAYPSHPMSYSTMLTQN

SLGNNHSFPASNGLSVDRLVNGEIPYATHHLTAAALAASVPCGLSVPCSGTYSLNPCSVNLLAGQTSYFF

PHVPHPSMTSQSSTSMSSRAASSSSPQTASSLPCDSLRPSLSSFSSGLSSGLSDYFTHQNQGSTSNPLIH

>DaRe_Foxg1b (NP_998079.2)

MGDQSEPTMVQKSTSFSIKSLLLPSKFDSADESAERGGSPAPVQDLDKPPEDAEMDNAQRDAEEPELQTK

KGKKFDKPPFSYNALIMMAIRQSPEKRLTLNGIYEFIMKNFPYYREHKQGWQNSIRHNLSLNKCFVKVPR

HYDDPGKGNYWMLDPSSDDVFIGGTTGKLRRRSATSRGKLVMKRGLRFAPLGLGLGERPSNPLYWQLSPF

LPLHHSHYNGSAHGFLNQGHTYGTLLPGVEPLGNGDMSRQILGASSGSINLSNGYGVSPPAAGLLSGHNG

YFVPGAQQPQSLPSAPGYGISSSQSPLLSDSLRTSLPSFTSPLSGGLLSQHKRVAPNSFLS

>DaRe_Foxg1c (NP_001038680.1)

MEDLKPPVSFFHKSSFSISSLLLRHERARSDAQEAPRSRSAKPPARCHQPADKPVRERNELVTRTEKKDG

VGEPKCEGTDVPEKKSKPDKPPFSYNALIMMAIRQSPERRLTLNGIYEFIMGNFPYYRENRQGWQNSIRH

NLSLNKCFVKVPRHYDDPGKGNYWMLDPSSDDVFIGGTTGKLRRRSTAASRAKLAMKRGARLSSTAASAG

LAFAGSFYWPVPPFVTLQHRHSSPAAAHHSNYAASVLSQSARHFSSVAPAAERLLIPSSQEATYYGMGCE

QMTSSSSSFSTSASVPLPLSAPCSFNLLSNQSSYFYSHQVPHTAGLSAWSQEESYLSKTSPSGQFFPGKP

SPSSSEYIGGLCTDIPSYFPHFNTASSMH

>DaRe_Foxg1’ (NP_001116096.1)

MEQKESPALQKLSSFSITSLLLPGKSGTPADSSPVTDPPSEERSSEKAKDAEDAGKPVKLDKPPFSYNAL

IMMAIRQSPEKRLTLNGIYEFIMKNFPFYREHKQGWQNSIRHNLSLNKCFVKVPRHYDDPGKGNYWMLDP

SSDDVFIGGTTGKLRRRSATSRGKLAIKRGLRFSPLGLHGITETANNPLYWQLSPLLSLHHHHHHPHYNG

TSHGFLNQAHGYGSFVHGVEHLGSREAPRAVLGGSSGALGLSNGYGMSSSPVGLLSVPSGLLPAPGLQSA

LGAPQSLRTALGPFTPTGAAALPALAHHDRLSPDSL

>MeCy_Foxg1 (XP_036399002.1)

MLDMGERKEVKMIPKSSFSINSLVPEAVQSDNHHRSAPEEEEKIPLPNPVQDGKSENICSKSDSSPESTC

ADEKEKQEEKKDCKEGDGGKDGEKKNGKYEKPPFSYNALIMMAIRQSPEKRLTLNGIYEFIMKNFPYYRE

NKQGWQNSIRHNLSLNKCFVKVPRHYDDPGKGNYWMLDPSSDDVFIGGTTGKLRRRSTTSRAKLAFKRGA

RLTSTGLTFMDRAGSLYWPMSPFLSLHHPRASSALSYNGTSSAYPSHPMSYSTMLTQNSLGNNHSFPASN

GLSVDRLVNGEIPYATHHLTAAALAASVPCGLSVPCSGTYSLNPCSVNLLAGQTSYFFPHVPHPSMTSQS

STSMTARAASSSSPQAPSSLPCESIRSSLPTFSSGLSGGLSDYFTHQNQGSTSNPLIH

>MeCy_Foxg1-like (XP_036403191.1)

MGDQKEPTMVHKSTSFSIKSLLLPSKCDKLESGAPEKNTLPAGSDSEKSLDPTEMDSTPFNQENQKEEDE

GSDRATEPSKNGKYDKPPFSYNALIMMAIRQSPEKRLTLNGIYEFIMKNFPYYREHKQGWQNSIRHNLSL

NKCFVKVPRHYDDPGKGNYWMLDPSSDDVFIGGTTGKLRRRSATSRGKLAMKRGLRFAPLGLGLNDRASN

PLYWQISPFLSLHHPHYNGSSPGFLNQGHAYGSLLSGVEQLGNGDLSRPILGGSTGGINLTNSYGVSTSP

VGLLSGQTGYFVSGTQHAQSLQQSGAGFAVPSSSPQTLISETLRTSLPSFTPGVSSGFSGVLSHQKRVPS

SFLN

> MeCy_Foxg1-like’ (XP_036373348.1)

MGDQKEPTMVHKSTSFSIKSLLFPSKYDNLGSGVPEKRTLPSGSDSEKSLDPTEMETGPFNQDTQKEGDE

GTGRPTEQSKNGKYDKPPFSYNALIMMAIRQSPEKRLTLNGIYEFIMKNFPYYREHKQGWQNSIRHNLSL

NKCFVKVPRHYDDPGKGNYWMLDPSSDDVFIGGTTGKLRRRSATSRGKLAMKRGLRFAPLSLGLGDRASN

PLYWQISPFLSLHHPHYNGSTPGFLNQGHAYGSLLSGVEQLGNGDLSRPVLGGSAGGISLSSGYGVSTSP

VGILSGHNGYFVSGTQHSQPLQSGAGYGVSCSSPSTLISETLRTSLPSFTPGVSTGFSGVLSHQKRVNSF

LT

>MeCy_Foxg1c (XP_036392434.1)

MFGMEDLKAPIRFFHKSSFSISSLLLRREGVMSDGGAVVEVPRSRSAKTRTHFLPGRTGRELCLRDEDYN

NADCNKNNDKTLCAVREERPVASEERRGKREERDGEGEERRGGEAERKVKYEKPPFSYNALIMMAIRQSP

ERRLTLSGIYEFIMGNFPYYRDNKQGWQNSIRHNLSLNKCFVKVPRHYDDPGKGNYWMLDPSSDDVFIGG

TTGKLRRRSTAASRAKLAIKRGTRLASTTAAGLAFTGSFYWPVAPFLTLQQPSQPHPGSALGYSSSYFGP

HHSNYASTVLSQTSQHISATVAGTDRLLQVTQETPFCGVTGGIPRRHQMSSSTSFAPTPLACALSLPNQC

SFNLLPSQASYIYSHQVPHPTTLGGLCPGQASPPKASPVGHFLTGRNGSSEYIGSLCAEFPNYCPQNNTT

TPLSAILP

>LaCh_Foxg1 (XP_006001628.1)

MLDMGDRKEVKMIPKSSFSINSLVPEAVQNDNHHRSAPEEDDKLVLPPLEPKNELVSTKGENPSAELTCT

EDKEKQEEKKAEGKEGETGKEGDKKNGKYEKPPFSYNALIMMAIRQSPEKRLTLNGIYEFIMKNFPYYRE

NKQGWQNSIRHNLSLNKCFVKVPRHYDDPGKGNYWMLDPSSDDVFIGGTTGKLRRRSTTSRAKLAFKRGA

RLTSTGLTFMDRAGSLYWPMSPFLSLHHPRASSTLSYNGTTSAYPSHPMSYSSVLTQNSLGNNHSFSTSN

GLSVDRLVNGEIPYATHHLTAAALAASVPCGLSVPCSGTYSLNPCSVNLLAGQTSYFFPHVPHPSMTSQS

STSMTARTASSSTSPPAPSTLPCESLRPSLPSFTTGLSGGLSDYFTHQNQGSSSNPLIH

>LaCh_Foxg1-like (XP_005991682.2)

MEDLKETKFVHKSSFSINSLLQEAVMSDRTIEREEEEKLLVTELECENSLLVVPAKTSLEDVREHEKEAE

KKEVEDSKEEKKEKHEKPPFSYNALIMMAIRQSPEKRLTLNGIYEFIMQNFPYYKENKQGWQNSIRHNLS

LNKCFVKVPRHYDDPGKGNYWMLDPSSDDVFIGGTTGKLRRRSTASRAKLAFKRGARLASSGLAFAGSLY

WPVSPFLSLHQPHSTSALGYSSSAYLTHPSSYASVFSQTSHQLNSSGVDRLLGTDTPYGSHHVTAAAFAS

SLPCGLSIPSSLSPCSFNLLAGQASYFFSQGPHHSMTGQSSSLASIHAASPKGTNDILGRPSPSFPRGIS

PDFSGFFPTQNQGASFNPILP

>LaCh_Foxg1-like’ (XP_014342331.1)

MGDQKEQKMVPKSTSFSIKSLLHPEAAPTDNCECKQEKQNASPETDPDPPEKKNGKLDKPPFSYNALIMM

AIRQSPEKRLTLNGIYEFIMKNFPYYRENKQGWQNSIRHNLSLNKCFVKVPRHYDDPGKGNYWMLDPSSD

DVFIGGTTGKLRRRSATSRGKLALKRGLRFAPLSLGLNDRASNPFYWQISPFLSLHHSHYNGASYLSHGA

GAYGSLLSGIEHYGVGDLARPVLGGSGTISVTNGGYSLSTSPVTLLSGQAGYFLSGAQHSQPIPHSASGF

GVPASPPQLLNSESVRPPGPLATFSSGLSSGFTGFLSQQSRTVSGSSFSH

>CrtCrt_Foxg1 (XP_048710341.1)

MLDMGDRKEGKMLPKSSFSINSLVPEAVQSDNNHSSHHPHHHNSHHPHPHHHHHHHHHHHPQQQQPQRAA

PAEEEEEEKSQLLLQPPVVAAAAAAAAAAATASGTLEVAKEILPGKGEPGSAAAAAELEEKEKAAAAAEE

KKGAAEGGKEGESGKEGEKKNGKYEKPPFSYNALIMMAIRQSPEKRLTLNGIYEFIMKNFPYYRENKQGW

QNSIRHNLSLNKCFVKVPRHYDDPGKGNYWMLDPSSDDVFIGGTTGKLRRRSTTSRAKLAFKRGARLTST

GLTFMDRAGSLYWPMSPFLSLHHPRASSTLSYNGTTSAYPSHPMPYSSVLTQNSLGNNHSFSTSNGLSVD

RLVNGEIPYATHHLTAAALAASVPCGLSVPCSGTYSLNPCSVNLLAGQTSYFFPHVPHPSMTSQSSTSMT

ARAASSSTSPQAPSTLPCESLRPSLPSFTTGLSGGLSDYFTHQNQGSSSNPLIH

>ChAb_Foxg1 (XP_032652532.1)

MLDMGDRKEVKMLPKSSFSINSLVPEAVQSDNNHNSHSHHHNSHHPHHHHHHHHHHHPQQQQQPQRAAPA

EEEEEKSQLLLQPPVVAAAAATTSGTLEVAKEILPGKGEPGSAAAAAELEEKEKAAEEKKGAAEGGKEGE

SGKEGEKKNGKYEKPPFSYNALIMMAIRQSPEKRLTLNGIYEFIMKNFPYYRENKQGWQNSIRHNLSLNK

CFVKVPRHYDDPGKGNYWMLDPSSDDVFIGGTTGKLRRRSTTSRAKLAFKRGARLTSTGLTFMDRAGSLY

WPMSPFLSLHHPRASSTLSYNGTTSAYPSHPMPYSSVLTQNSLGNNHSFSASNGLSVDRLVNGEIPYATH

HLTAAALAASVPCGLSVPCSGTYSLNPCSVNLLAGQTSYFFPHVPHPSMTSQSSTSMTARAASSSTSPQA

PSTLPCESLRPSLPSFTTGLSGGLSDYFTHQNQGSSSNPLIH

>ChAb_Foxg1-like (XP_032653905.1)

MASSRGGAGEDRSAAAAARGGGELEKGRGEAPGKQHDKPPFSYNALIMMAIRQSPGRRLTLNGIYEFIMR

NFPYYRENRQGWQNSIRHNLSLNKCFVKVPRHYDDPGKGSYWTLHPSSEDVFIGGTTGKLRRRAPTGQAK

VAFRRGAGVAFASALYWPLPPFLALPQQPTPTAGYGSYSGSYSSAFCPGADGLFGGGDVPYGGHHQQVTA

GSSLSCGFSAPSTFNSFNLLAGQASYILAQRPQPAPAGTSLFPGYLASLNQGSPFNPALP

>GoEv_Foxg1 (XP_030418155.1)

MLDMGDRKEVKMLPKSSFSINSLVPEAVQSDNNHNSHSHHHNSHHPHHHHHHHHHHHPQQQQPQRAAPAE

EEEEKSQLLLQPPVVAAAAAAAATTSGTLEVAKEILPGKGEPGSAATAAELEEKEKAAEEKKGAAEGGKE

GESGKEGEKKNGKYEKPPFSYNALIMMAIRQSPEKRLTLNGIYEFIMKNFPYYRENKQGWQNSIRHNLSL

NKCFVKVPRHYDDPGKGNYWMLDPSSDDVFIGGTTGKLRRRSTTSRAKLAFKRGARLTSTGLTFMDRAGS

LYWPMSPFLSLHHPRASSTLSYNGTTSAYPSHPMPYSSVLTQNSLGNNHSFSTSNGLSVDRLVNGEIPYA

THHLTAAALAASVPCGLSVPCSGTYSLNPCSVNLLAGQTSYFFPHVPHPSMTSQSSTSMTARAASSSTSP

QAPSTLPCESLRPSLPSFTTGLSGGLSDYFTHQNQGSSSNPLIH

> GoEv_Foxg1-like (XP_030400210.1)

MMAIRQSPGRRLTLNGIYEFIMRNFPYYRENRQGWQNSIRHNLSLNKCFVKVPRHYDDPGKGSYWTLHPS

SEDVFIGGTTGKLRRRAPAGRAKVAFRRGAGVAFASSLYWPLPPFLALPQQPTPTASYGSYSGSYSSAFC

PGADRLFGGGNVPYGGHCQQVTAASSLSSGFSAPSTLNSFNLLAGQASYILAQRPQPAPAGASLFPGYLA

SLNQGSPFNPALP

>PyBi_Foxg1 (XP_007436149.1)

MLDMGDRKEVKMLPKSSFSINSLVPEAVQSDNNHSHQHHPHHHNNHHPHHHHHHHHHHHHPAQPPQPQRA

TPAEDDEEEEKSQLLLQPPGVGAAAAAELDDKEKGPSEEKKGVSEGAKEAGGGDGGGKEGEKKNGKYEKP

PFSYNALIMMAIRQSPEKRLTLNGIYEFIMKNFPYYRENKQGWQNSIRHNLSLNKCFVKVPRHYDDPGKG

NYWMLDPSSDDVFIGGTTGKLRRRSTTSRAKLAFKRGARLTSTGLTFMDRAGSLYWPMSPFLSLHHPRAS

STLSYNGSTSAYPSHPMPYSSVLTQNSLGNNHSFSASNGLSVDRLVNGELPYATHHLTAAALAASVPCGL

SVPCSGTYSLNPCSVNLLAAGQTSYFFPHVPHPSMTSQSSSSMTGRAASSSTSPQAPSTLPCESLRPSLP

SFTTGLSGGLSDYFTHQNQGSSSNPLIH

> PyBi_Foxg1-like (XP_025029727.1)

MEQFTPNSWVLKSPFSITNLLQDVAASKDLEGSLSSLLGEEEVVAVPSKGCQTLRRDISAERRGKPEKPP

FSYNALIVMAIRQSPEKRLTLSGIYEFIMGNFPYYQENKQGWQNSVRHNLSLNKCFVKVPRHYDDPGKGN

YWILDSSSEDISIGGTAGKLKRKTVASRTKMAIWKGARLPSTGMTLDGSFHLPLPPLYGVTSAYLGHPSS

SFSQQALTPPRRHRQIERGSHQHLVATMLASTLPYSLIRSDHLDSCSVNFLEGQASSTFSWRHHHTLPMN

SQFGLPSKSIGNLLGGVLPTFPGQLSPEFPTFFAFQNHQASFKPSLL

>AnCa_Foxg1 (XP_003214519.1)

MLDMGDRKEVKMLPKSSFSINSLVPEAVQNDNGSSSSSNHNHGHQHHHNNHHPHHHHHHHHHHIHQQQQQ

APQRGAPTAEEDEEEDKGQLLLQPPGAGGATSGAPSSETVVSKGDPVSDLEGEKEKGSPEEKKASPDGAK

DSEGGKEGEKKNGKYEKPPFSYNALIMMAIRQSPEKRLTLNGIYEFIMKNFPYYRENKQGWQNSIRHNLS

LNKCFVKVPRHYDDPGKGNYWMLDPSSDDVFIGGTTGKLRRRSTTSRAKLAFKRGARLTSTGLTFMDRAG

SLYWPMSPFLSLHHPRAGGALGYNSAASAYPSHPMPYSSVLAQNSLSNQHSFAPSNGLSVDRLVNGELPY

ATHHLTAAAALAASVPCGLSVPCSGATYSLNPCSVNLLAAGQTSYFFPHVPHPSMSSSSSSSQSSSSASL

AAVGRAASASSSPQAPSSASGPLSCEALRPTLPPAFSTGLSGGLSDYFTHQNQGASSNPLIH

>AnCa_Foxg1-like (XP_016853233.1)

MEGLNANSWVLKSPFSITNLLQDVASSDVPRGPLSSSSSSSPSSMGDEAVEGHPKTRPGERRQGKEEKPP

FSYNALIMMAIRQSPGKRLTLNGIYEFIMRSFPYYKENKQGWQNSIRHNLSLNKCFVKVPRHYDDPGKGN

YWMLDPSSEEDVFIGGATGKLRRRSPSSQAKLAFRRGQRLSSAGVTLSGSFYWPFPPFYGTSSAFLGHHQ

PGSFSSTFPHQVPTPSGIQRQMDGAEDPYGSHQHVAATMLASAVPRCLSAAEPLDSYSVNFVAGQTSYVL

SPRHTLPFGFQAGLLSKSTGSVFHGGPLAFPGQLSPEAPSYFPFQNHGAPFHP

>PlWa_HypotheticalProtein (KAJ1103213.1)

MLDMGERKEVKMIPKSSFSINSLVPEAVQNDNHPPAPHHHHPHHHHHHPHQHHSQHHQHPPPPGPHHRPE

DEPADKSLPEPAAPELPPVEPEDKAAAEEKKGDAGGGKEGEGPKEGDKKNGKYEKPPFSYNALIMMAIRQ

SAEKRLTLNGIYEFIMKNFPYYRENKQGWQNSIRHNLSLNKCFVKVPRHYDDPGKGNYWMLDPSSDDVFI

GGTTGKLRRRSTTSRAKLAFKRGARLASGGLTFMDRAGSLYWPMSPFLSLHHPRASGGLGYGGGASAYPG

HPVPYSSVLAQNSLGGGHSFSAAANGLSVDRLVNGELPYAAHHLTAAALAASVPCGLSVPCSGTYSLNPC

SVNLLTAGQTSYFFPHVPHPSMTAQGGAAMQARAASSASSSPQAPSSLACEALRPSLPSFSSGLSGGLSD

YFTHQPQGSSSNPLIH

>PlWa_HypotheticalProtein’ (KAJ1107001.1)

MGAEGSLGACDQPREEEKVPEEPRDGKGDEGQEKHSKPPFSYNALIMMAIRGSPGRRLTLSGIYEYIMEN

FPYYRDNRPGWQNSIRHNLSLNKCFLKVPRHYDDPGKGNYWVLDPSSDDVFIGGTTGKLRRRTTGSRAKL

AFRRGGRAASSGLTLTGSLYWPLGPFLSIHPPPQAYSAPLGCSSSSPTCFSHPTSCAAMLSQAARTLGAP

GLERFIPADASCGHHHVTATLTPSLPCGVPSSLNSCSFSMLSGQANYYYCQRGLHQPSLPPLPSPYTKTT

FDFPGRPPQQFPTGLSQDCPYLFTPHNETPSFHPGMP

>XeLa_Foxg1 (NP_001079165.1)

MLDMGDRKEVKMIPKSSFSINSLMPEAVQNDNHPQPHHHHHHQQQPQHLQLPQQHHLQPHHRPLQEEDEL

DKSLLEVKTESLPPGKGDPAASELPGEDKDKIDDKKVDGKDGDSGKDGGDKKNGKYEKPPFSYNALIMMA

IRQSPEKRLTLNGIYEFIMKNFPYYRENKQGWQNSIRHNLSLNKCFVKVPRHYDDPGKGNYWMLDPSSDD

VFIGGTTGKLRRRSTTSRAKLAFKRGARLTSTGLTFMDRAGSLYWPMSPFLSLHHPRASSTLSYNGTTSA

YPSQPMPYSSVLTQNSLGNNHSFSTSNGLSVDRLVNGEIPYATHHLTAAALAASVPCGLPVPCSGTYSLN

PCSVNLLAGQTGYFFPHVPHPSITSQSSTSMAARAASSSTSPQAPSTLPCESLRPALPSFTTGLSGGLSD

YFTHQNQGSSSNSLIH

>GaGa_Foxg1 (NP_990524.1)

MLDMGDRKEVKMLPKSSFSINSLVPEAVQSDNHSGHSHHNSHHPHHHHHHHHHHPPPPQQPQRAAAAEEE

DEEKAPLLLPPPAAGALEAAKAEALAGKGEAGAAAAELEEKEKAAEEKKGAAEGGKDGESGKEGEKKNGK

YEKPPFSYNALIMMAIRQSPEKRLTLNGIYEFIMKNFPYYRENKQGWQNSIRHNLSLNKCFVKVPRHYDD

PGKGNYWMLDPSSDDVFIGGTTGKLRRRSTTSRAKLAFKRGARLTSTGLTFMDRAGSLYWPMSPFLSLHH

PRASSTLSYNGTASAYPSHPMPYSSVLTQNSLGNNHSFSTSNGLSVDRLVNGEIPYATHHLTAAALAASV

PCGLSVPCSGTYSLNPCSVNLLAGQTSYFFPHVPHPSMTSQSSTSMTARAASSSTSPQAPSTLPCESLRP

SLPSFTTGLSGGLSDYFTHQNQGSSSNPLIH

>VoUr_Foxg1 (XP_027714376.1)

MLDMGDRKEVKMIPKSSFSINSLVPEAVQSDNHHAGHGHHNSHHPPPHHHHHHGHHHHHHPPPPPPPQQQ

QPPQQQQQQQHPPPPQPPQAPQARSAPADDDEDKGPQQLLLPPPPQGAASAATASAALDGAKADGLGGKG

EPGGGGGGGAGDLAPVGQEEKEKGSGGEEKKGAGEGGKDGEGGKEGEKKNGKYEKPPFSYNALIMMAIRQ

SPEKRLTLNGIYEFIMKNFPYYRENKQGWQNSIRHNLSLNKCFVKVPRHYDDPGKGNYWMLDPSSDDVFI

GGTTGKLRRRSTTSRAKLAFKRGARLTSTGLTFMDRAGSLYWPMSPFLSLHHPRASSTLSYNGTTSAYPS

HPMPYSSVLTQNSLGNNHSFSTSNGLSVDRLVNGEIPYATHHLTAAALAASVPCGLSVPCSGTYSLNPCS

VNLLAGQTSYFFPHVPHPSMTSQSSTSMTARAASSSTSPQAPSTLPCESLRPSLPSFTTGLSGGLSDYFT

HQNQGSSSNPLIH

>MuMu_Foxg1 (NP_001153584.1)

MLDMGDRKEVKMIPKSSFSINSLVPEAVQNDNHHASHGHHNSHHPQHHHHHHHHHHPPPPAPQPPPPPPQ

QQQQQPPPAPQPPQARGAPAADDDKGPQPLLLPPSTALDGAKADALGAKGEPGGGPAELAPVGPDEKEKG

AGAGGEEKKGAGEGGKDGEGGKEGDKKNGKYEKPPFSYNALIMMAIRQSPEKRLTLNGIYEFIMKNFPYY

RENKQGWQNSIRHNLSLNKCFVKVPRHYDDPGKGNYWMLDPSSDDVFIGGTTGKLRRRSTTSRAKLAFKR

GARLTSTGLTFMDRAGSLYWPMSPFLSLHHPRASSTLSYNGTTSAYPSHPMPYSSVLTQNSLGNNHSFST

ANGLSVDRLVNGEIPYATHHLTAAALAASVPCGLSVPCSGTYSLNPCSVNLLAGQTSYFFPHVPHPSMTS

QTSTSMSARAASSSTSPQAPSTLPCESLRPSLPSFTTGLSGGLSDYFTHQNQGSSSNPLIH

>HoSa_FOXG1 (NP_005240.3)

MLDMGDRKEVKMIPKSSFSINSLVPEAVQNDNHHASHGHHNSHHPQHHHHHHHHHHHPPPPAPQPPPPPQ

QQQPPPPPPPAPQPPQTRGAPAADDDKGPQQLLLPPPPPPPPAAALDGAKADGLGGKGEPGGGPGELAPV

GPDEKEKGAGAGGEEKKGAGEGGKDGEGGKEGEKKNGKYEKPPFSYNALIMMAIRQSPEKRLTLNGIYEF

IMKNFPYYRENKQGWQNSIRHNLSLNKCFVKVPRHYDDPGKGNYWMLDPSSDDVFIGGTTGKLRRRSTTS

RAKLAFKRGARLTSTGLTFMDRAGSLYWPMSPFLSLHHPRASSTLSYNGTTSAYPSHPMPYSSVLTQNSL

GNNHSFSTANGLSVDRLVNGEIPYATHHLTAAALAASVPCGLSVPCSGTYSLNPCSVNLLAGQTSYFFPH

VPHPSMTSQSSTSMSARAASSSTSPQAPSTLPCESLRPSLPSFTTGLSGGLSDYFTHQNQGSSSNPLIH
